# Incorporation of a Nitric Oxide Donating Motif into Novel PC-PLC Inhibitors Provides Enhanced Anti-Proliferative Activity

**DOI:** 10.3390/ijms222111518

**Published:** 2021-10-26

**Authors:** Shaun W. P. Rees, Tayla A. Rees, Euphemia Leung, Christopher S. Walker, David Barker, Lisa I. Pilkington

**Affiliations:** 1 School of Chemical Sciences, University of Auckland, Auckland 1010, New Zealand; sree168@aucklanduni.ac.nz; 2 School of Biological Science, University of Auckland, Auckland 1010, New Zealand; tree533@aucklanduni.ac.nz (T.A.R.); cs.walker@auckland.ac.nz (C.S.W.); 3 Maurice Wilkins Centre for Molecular Biodiscovery, University of Auckland, Auckland 1010, New Zealand; e.leung@auckland.ac.nz; 4 Auckland Cancer Society Research Centre, University of Auckland, Grafton, Auckland 1023, New Zealand; 5 MacDiarmid Institute for Advanced Materials and Nanotechnology, Wellington 6012, New Zealand

**Keywords:** 2-morpholinobenzoic acids, anti-proliferative activity, DAF-FM, NF-κB, nitric oxide donors, PC-PLC, triple-negative breast cancer

## Abstract

Inhibition of phosphatidylcholine-specific phospholipase C (PC-PLC) has previously been shown to be a potential target for novel cancer therapeutics. One downstream consequence of PC-PLC activity is the activation of NF-κB, a nuclear transcription factor responsible for transcribing genes related to oncogenic traits, such as proliferation, angiogenesis, metastasis, and cancer cell survival. Another biological pathway linked to NF-κB is the exogenous delivery of nitric oxide (NO), which decreases NF-κB activity through an apparent negative-feedback loop. In this study, we designed and synthesised 13 novel NO-releasing derivatives of our previously reported class of PC-PLC inhibitors, 2-morpholinobenzoic acids. These molecules contained a secondary benzylamine group, which was readily nitrosylated and subsequently confirmed to release NO in vitro using a DAF-FM fluorescence-based assay. It was then discovered that these NO-releasing derivatives possessed significantly improved anti-proliferative activity in both MDA-MB-231 and HCT116 cancer cell lines compared to their non-nitrosylated parent compounds. These results confirmed that the inclusion of an exogenous NO-releasing functional group onto a known PC-PLC inhibitor enhances anti-proliferative activity and that this relationship can be exploited in order to further improve the anti-proliferative activity of current/future PC-PLC inhibitors.

## 1. Introduction

Phosphatidylcholine-specific phospholipase C (PC-PLC) is a key regulatory enzyme that is responsible for hydrolysis of the abundant phospholipid, phosphatidylcholine, into secondary messengers diacylglycerol (DAG) and phosphocholine (PCho) [1,2]. PC-PLC metabolites DAG and PCho have been implicated in various oncogenic pathways, such as proliferation, differentiation, cell-cycle activation, and increased cell motility, across multiple cancer subtypes [2,3,4]. The specific role of PCho in these processes has yet to be fully clarified despite there being clear evidence of elevated concentrations of PCho in a range of common cancer subtypes [5,6]. The role of DAG in these oncogenic processes has been established to be through activation of protein kinase C enzymes (PKC), leading to the induction of mitogenesis via phosphorylation of various substrates [6,7]. A well-known effect of PKC activity is the activation of transcription factor NF-κB, resulting in transcription of various genes involved in cancer cell survival, proliferation, angiogenesis, and metastasis [5,6,7,8,9,10,11]. Furthermore, treatment with PC-PLC inhibitor tricyclodecan-9-yl-potassium xanthate (D609) has been shown to prevent activation of NF-κB in mammalian cells, further substantiating a direct link between PC-PLC and NF-κB [11,12,13]. 

The effect of PC-PLC inhibition has been investigated in various cancer cell lines to determine its viability as a novel target for cancer treatment. In the triple-negative breast cancer (TNBC) cell line MDA-MB-231, where PC-PLC was expressed at two- to six-fold higher concentrations than basal levels, treatment with D609 halted proliferation, caused progressive reversal of mesenchymal traits, and reduced motility of the cancer cells [4]. There is also evidence of PC-PLC inhibition halting cell-cycle progression in epithelial ovarian cancer cells, where PC-PLC expression is three- to five-fold higher than basal levels [3]. Reduction in cell-cycle progression was also reported in the rat hepatocellular cell line CBRH-7919, following treatment with D609 [4]. Overall, these results demonstrate PC-PLC to be a viable target for novel cancer treatments due to the apparent anti-cancer efficacy displayed by D609.

Another enzyme of note related to the PC-PLC/PKC/NF-κB pathway is inducible nitric oxide synthase (iNOS). There are three members of the nitric oxide synthase (NOS) family, endothelial NOS (eNOS), neuronal NOS (nNOS), and inducible NOS (iNOS) [14]. Both eNOS and nNOS activation is dependent on an increase in calcium ion concentrations, and both enzymes are only capable of producing nitric oxide (NO) at nanomolar levels on a second-minute time scale when activated [14,15]. In contrast, iNOS expression is independent of Ca^2+^ concentration and is able to generate NO in the micromolar range for hours and, in some cases, days [15]. Rather than Ca^2+^, iNOS expression is activated by inflammatory pathways, one example being NF-κB, which is known to have multiple binding sites on the promotor region of the iNOS gene [14,15]. iNOS and PC-PLC seem to also be linked, as PC-PLC stimulation has been illustrated to increase iNOS transcription via NF-κB activity [8]. Furthermore, inhibition at each of the three levels of the PC-PLC/PKC/NF-κB pathway led to decreased iNOS expression, further solidifying the connection between these cellular mechanisms [16,17,18]. 

Of more interest to this study, however, is the well-established negative feedback loop between NO and NF-κB activity [15,19,20,21]. It has been observed that exogenous NO, when administered via NO donors, such as *S*-nitroso-*N*-acetylpenicillamine (SNAP) and sodium nitroprusside (SNP), causes inhibition of NF-κB activity, halting NF-κB translocation to the nucleus, thereby preventing NF-κB producing a mitogenic signal [19,20,21]. This negative feedback mechanism suggests that if exogenous NO administration is able to be combined with a PC-PLC inhibitor, it may blockade the PLC/PKC/NF-κB pathway through two different modes of inhibition, leading to greater efficacy of the anti-proliferative treatment.

**Figure 1:**
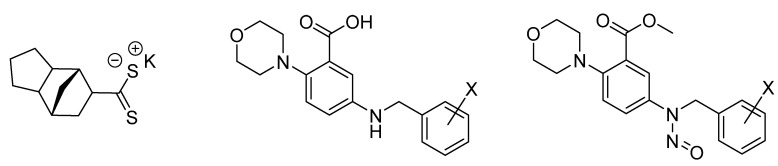
Structures of D609 (**left**) and 2-morpholinobenzoic acids (**centre**) compared to *N*-nitrosylated benzylamines (**right**) investigated in this study. X = H, F, Cl, Br, OMe.

Despite this link between PC-PLC inhibition and exogenous NO, a single molecule with both functionalities has yet to be reported in the literature. We have, however, recently reported the discovery of a novel class of PC-PLC inhibitors, the 2-morpholinobenzoic acids, which are able to inhibit PC-PLC at superior levels to the aforementioned literature standard D609 (Figure 1) [22]. Furthermore, these molecules possess a secondary benzylamine group, which has the ability to act as a substrate for *N*-nitrosylation, allowing possible incorporation of an NO group into this class of potent PC-PLC inhibitors.

In this study, we aimed to synthesise a singular class of molecules with both PC-PLC inhibitory activity and NO donating functionality, in order to determine if these pathways would combine to produce enhanced anti-proliferative activity when compared to PC-PLC inhibition alone. It was expected that under physiological conditions, the labile N-NO bond would be cleaved, releasing NO whilst also giving a 2-morpholinobenzoic acid derivative, which we have shown inhibits PC-PLC. The 2-morpholinobenzoic acid derivatives with methyl ester groups were chosen for this study, as they have already been shown to induce moderate anti-proliferative efficacy though PC-PLC inhibition alone [23] and therefore making it possible to elucidate if incorporation of a NO-releasing group provides the desired increase in anti-proliferative activity through increased inhibition of NF-κB in a dual-action mechanism.

## 2. Results and Discussion

### 2.1. Synthesis of N-Nitrosylated Benzylamines

**Scheme 1:**
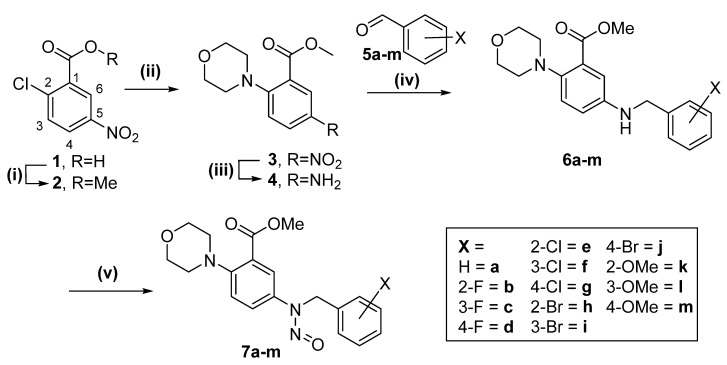
Synthesis of desired *N*-nitrosylated methyl esters **7a**–**m**. Reagents and conditions: (**i**) H_2_SO_4_ (cat.), MeOH, 80 °C, 24 h, 96%; (**ii**) morpholine (3 eq.), MeCN, r.t., 24 h, 99%; (**iii**) Pd/C (10% w/w), H_2_ (1 atm.), EtOAc, r.t., 24 h, 97%; (**iv**) (Step 1) **5a**–**m** (2 eq.), MeOH, reflux, 24 h; (Step 2) NaBH_4_ (3 eq.), MeOH/THF (2:3), 35 °C, 24–72 h (53–95%, over two steps); (**v**) NaNO_2_ (1.05 eq.), *p*-TsOH (1.05 eq.), CH_2_Cl_2_, r.t., 24 h (63–94%).

In order to synthesize the desired *N*-nitrosylated compounds, we first had to synthesize benzylamines **6a**–**m** from 2-chloro-5-nitrobenzoic acid, **1.** This was achieved using previously reported methods (Scheme 1) [22,23]. First, the carboxylic acid motif on compound **1** was converted into the methyl ester on compound **2**, and then, the 2-Cl group was substituted with a morpholine ring affording **3**. Next, the 5-nitro group was reduced via catalytic hydrogenation to an amine, yielding aniline **4**. Following this, aniline **4** was subjected to a two-pot reductive amination with various benzaldehydes **5a**–**m**, producing aforementioned benzylamines **6a**–**m**. A range of benzaldehydes **5a**–**m** with F, Cl, Br, and OMe substitution at the 2-, 3- and 4- positions around the aromatic ring were selected, as these substituents provided the best PC-PLC inhibitory activity for the carboxylic acid series [22]. Benzylamines **6a**–**m** were then nitrosylated using sodium nitrite and *p*-toluenesulfonic acid in dichloromethane affording the desired novel *N*-nitrosylated benzylamines **7a**–**m** in good to excellent yields [24]. It was noted that there was no discernible trend relating reaction yield and substitution position. Compounds **7a**–**m** were analysed using Lipinski’s rule-of-five and were all found to adhere to these parameters, suggesting these compounds display the required pharmacokinetics to be considered drug-like (Table S1) [25]. 

### 2.2. Assessment of NO Donor Activity by N-Nitrosylated Benzylamines

Following the synthesis of *N*-nitrosylated benzylamines **7a**–**m**, the ability for these compounds to release NO in vitro was analysed using a method based on a highly-sensitive fluorescence assay reported by Tjalkens et al. [26]. Each compound **7a**–**m** was incubated for 2 h with HEK293S cells pretreated with the NO activated fluorophore DAF-FM. The resulting mean fluorescent intensity (F) was measured and quantified as a ratio, F/F_0_, with F_0_ being the mean baseline fluorescence for each molecule. Compound **6a** was also included in the fluorescence assay as a negative control due to its lack of a secondary nitrosamine group.

**Figure 2:**
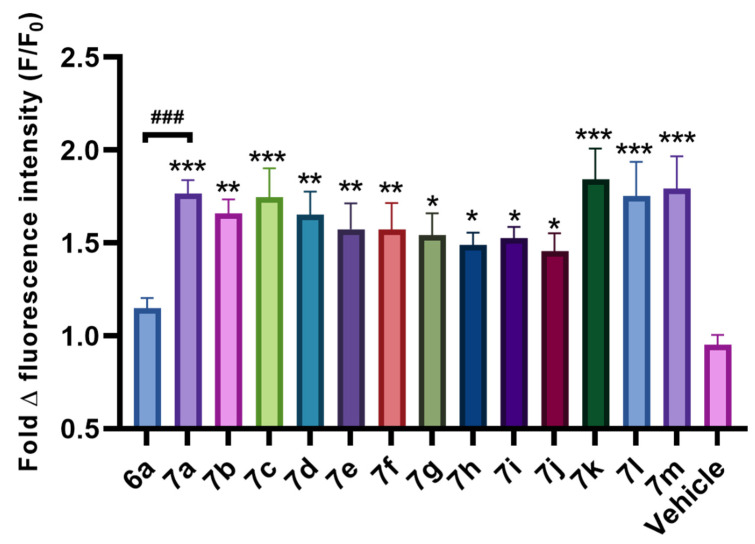
Compounds donate NO to DAM-FM in HEK293S cells. HEK293S cells were treated with 100 µM of each compound and incubated for 2 h. NO release was detected using DAF-FM and fluorescently imaged in the fluorescein channel. NO release was quantified using Perkin Elmer Columbus software by comparing mean fluorescence intensity prior to and 2 h after the addition of compound. Data are expressed at mean ± s.e.m of five independent experiments in triplicate. * *p* < 0.05, ** *p* < 0.01, *** *p* < 0.001 compared to vehicle by one-way ANOVA with a post-hoc Dunnett’s test. ### < 0.001 for **6a** and **7a** when compared by Student’s unpaired *t*-test. The mean of each compound (**7a**–**m**) was compared to each other by one-way ANOVA with post-hoc Tukey’s multiple comparisons test; no significant differences were observed.

**Figure 3:**
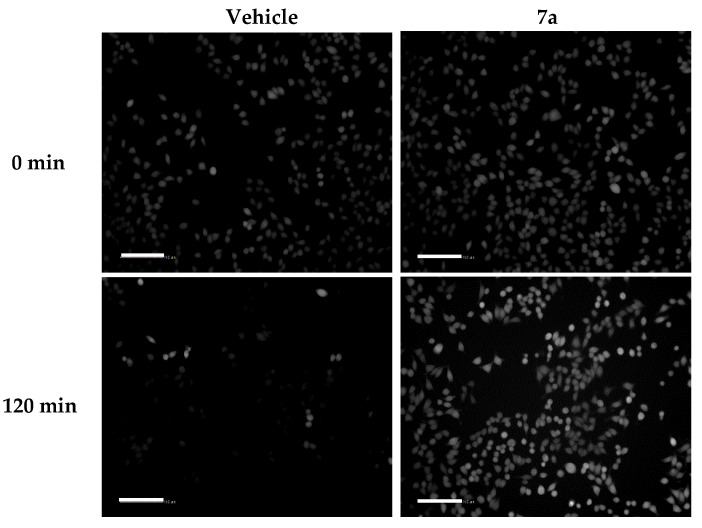
Changes in DAF-FM fluorescence intensity in response to compound **7a** and vehicle (DMSO). Images are representative from five independent experiments, scale bars = 100 µm. DAF-FM fluorescence is shown in grey scale; contrast and brightness were uniformly adjusted and applied for each compound over the two time periods. Images were taken at 20× high numerical aperture (0.7 NA) on Perkin Elmer Operetta.

Pleasingly, all *N*-nitrosylated benzylamines **7a**–**m** displayed a 1.5- to 2-fold increase in mean fluorescence intensity, which was a significant increase (*p*-value < 0.05) compared to vehicle, suggesting that the secondary nitrosamine group was able to release NO (Figure 2). Using images taken of the cells following two hour incubation (Figure 3), it is clear that *N*-nitrosylated **7a** displayed a marked increase in fluorescence when compared to the vehicle. The results suggested that the electron donating methoxy substituents (**7k**–**m**) elicited marginally better NO release, followed by the unsubstituted benzylic ring (**7a**) and finally the electron-withdrawing halogens (**7b**–**7j**). However, these substituent-related observations were not statistically different; therefore, it cannot be concluded that altering the electron density of the *N*-benzyl group had a definitive effect on NO release.

Importantly, non-nitrosylated compound **6a** illustrated only a negligible (non-significant) increase in fluorescence compared to the vehicle. This indicates that the increased fluorescence demonstrated by **7a**–**m** is a function of NO release from the nitroso moiety and not a function of the underlying molecular scaffold, therefore confirming that the secondary nitrosamine group was successfully releasing NO. 

### 2.3. Anti-Proliferative Activity of N-Nitrosylated Benzylamines

In order to assess the anti-proliferative activity of *N*-nitrosylated benzylamines **7a**–**m**, a ^3^H thymidine incorporation assay was carried out using MDA-MB-231 and HCT116 cancer cell lines. The values provided were then compared to the anti-proliferative activity of non nitrosylated compounds **6a**–**m** in order to ascertain if the inclusion of NO donating functionality improved the anti-proliferative abilities of these compounds.

As previously reported, non-nitrosylated benzylamines **6a**–**m** possess poor-to-moderate anti-proliferative activity against triple negative breast cancer MDA-MB-231 cells, with the best result being **6h**, which reduced growth to 67% of positive control [23]. In contrast, twelve of the thirteen *N*-nitrosylated benzylamines **7a**–**m** displayed significantly (*p*-value < 0.05) improved anti-proliferative activity compared to their non-nitrosylated counterparts (Table 1). Additionally, seven of the thirteen *N*-nitrosylated benzylamines reduced MDA-MB-231 cell growth to less than 40%, with the most potent compounds being the brominated analogues **7h**, **7i** and **7j**, which decreased cell growth to 4%, 24.5%, and 18.8%, respectively. 

The trend relating benzylic substitution and anti-proliferative activity was shared between the *N*-nitrosylated series **7a**–**m** and their parent series **6a**–**m**, with the brominated compounds **h**, **i,** and **j** being the most active, followed by chlorinated compounds, **e**, **f**, and **g**. The consistency of this trend suggests that a similar PC-PLC inhibitory effect is maintained by these compounds following in-vitro NO release, indicating that the anti-proliferative activity is likely due to synergism between NO release and PC-PLC activity rather than through NO release alone.

*N*-Nitrosylated benzylamines **7a**–**m** also displayed greatly improved anti-proliferative activity in the human colon cancer cell line HCT116, a cell line in which benzylamines **6a**–**m** had previously shown little to no activity (Table 2). It was discovered that twelve of the thirteen *N*-nitrosylated benzylamines demonstrated significantly (*p*-value < 0.05) better anti-proliferative actions compared to the poor activity of their corresponding non-nitrosylated parents **6a**–**m**. Again, it was the brominated **7h**–**j** and chlorinated **7e**–**g** compounds that demonstrated the best activity, with the 2-Br analogue **7h** being the standout, decreasing growth by ~75%. It can be seen that in general the tested compounds were more effective against MDA-MB-231 than HCT116, which was expected due to the known high expression of PC-PLC in the MDA-MB-231 cell line [4]. 

Overall, these results demonstrate that the inclusion of a NO releasing motif onto a known PC-PLC inhibitor significantly increased anti-proliferative activity in both triple-negative breast cancer and colon cancer cell lines when compared to their non-nitrosylated counterparts acting through PC-PLC inhibition alone. These results suggests that both the NO releasing and PC-PLC inhibitory mechanisms are working together, potentially through a dual-action inhibition of the PC-PLC/PKC/NF-κB pathway in order to enhance the overall anti-proliferative ability of this class of compounds.

## 3. Materials and Methods

### 3.1. Synthesis of N-Nitrosylated Derivatives

#### 3.1.1. Synthesis of Compounds **2**–**6a**–**m**

Compounds **2**–**6a**–**m** were synthesized in accordance with our previously reported procedures [22,23].

#### 3.1.2. General Procedure for Synthesis of N-Nitrosylated Benzylamines **7a**–**m**

To a stirred solution of **6a**–**m** (1 equiv.) and NaNO_2_ (1.05 equiv.) in anhydrous CH_2_Cl_2_ (5 mL) was added *p*-toluenesulfonic acid monohydrate (1.05 equiv.) portion-wise over 5 min, and the mixture then was allowed to stir overnight at room temperature. Following this, the reaction mixture was filtered and the organic filtrate washed with water (3 × 10 mL), brine (10 mL), and dried with MgSO_4_. This suspension was filtered, and the filtrate was evaporated in vacuo to give the crude product as a red oil, which was purified with flash chromatography to give the desired *N*-nitrosylated benzylamine **7a**–**m.**

### 3.2. Nitric Oxide Release Assay

#### 3.2.1. Cell Culture

HEK293S cells were cultured as previously described [27]. Briefly, cells were cultured in high-glucose DMEM (11965092, Thermo-Fisher, Waltham, MA, USA) supplemented with 8% foetal bovine serum (Life Technologies, Carlsbad, CA, USA) in a 37 °C humidified incubator at 5% CO_2_. HEK293S cells were plated into 96-well cell-carrier ultra plates (PerkinElmer, Waltham, MA, USA) pre-coated with poly-D-lysine at a density of 8000 cells per a well. Forty-eight hours after plating, cells were used for live cell-imaging NO experiments.

#### 3.2.2. Intracellular NO Release and Detection

The generation of NO in HEK293S cells was detected by a 3-amino-4-aminomethyl-2′,7′-difluorescein diacetate (DAF-FM) probe. Cells were pre-treated with 5 µM of DAF-FM in assay media and HBSS (Gibco) with 10 mM HEPES (Gibco) for 30 min at 37 °C. Media was then discarded, cells were washed once with assay media, and then incubated in fresh assay media for 1 h at 37 °C. Cells were then imaged on an Operetta high-content imaging system using a 20× high NA objective (NA 0.7) (PerkinElmer) to obtain the baseline fluorescence. Cells were then treated with 100 µM of compound or vehicle alone in triplicate for 2 h and incubated at 37 °C. NB: Cells were treated with this concentration of the compound to improve the signal to noise ratio to allow the fluorescence changes to be accurately measured. It should be noted that DAF-FM is a fluorescein-based fluorophore that emits at a similar wavelength to cell auto-fluorescence from endogenous fluorophores, such as flavin and flavoproteins [26,28]. Additionally, the higher concentration allows the differentiation between the NO released from the compound and much slower endogenous release. Cells were then imaged again on the Operetta and fold-change in fluorescence intensity was calculated using the baseline intensity. Image analysis was performed on unprocessed images using Columbus (PerkinElmer). All data are mean ± standard error of the mean (s.e.m) combined from five independent experiments and were analysed using Prism GraphPad 8.0.2. Mean fold change in fluorescence intensity was analysed using one-way ANOVA with post-hoc Dunnett’s test.

### 3.3. Cell Proliferation Assay

The colorectal cancer cell line HCT116 and triple-negative breast cancer cell line MDA-MB-231 were purchased from the ATCC, grown in alpha-MEM containing 5% foetal calf serum (FCS), penicillin/streptomycin (100 U/mL and 100 µg/mL, respectively), and insulin/transferrin/selenium supplement (Roche). Inhibition of cell proliferation for compounds **6a**–**m** has previously been reported [23]. Cell proliferation assays for **7a**–**m** were conducted according to literature methods [23,29]. Briefly, 3000 cells per well were seeded into 96-well plates, which were tissue culture-treated for monolayer culture and incubated in the presence of 10 μM of 7a-m for 3 days. Prior to harvest, 0.04 μCi of ^3^H-thymidine was added to each well and incubated for 5 h, after which the cells were transferred using an automated TomTec harvester onto glass fibre filters. Filters were incubated with Betaplate Scint, and incorporation of radiolabeled thymidine was measured using a Trilux/Betaplate counter. All experiments were performed using triplicate wells, repeated three times, and reported as mean ± standard error of the mean. 

## 4. Conclusions

PC-PLC inhibition and exogenous NO delivery have both been shown to be decrease the activity of NF-κB, a transcription factor involved in the regulation of various genes associated with cancer cell survival, proliferation, angiogenesis, and metastasis [8,9,10,11,12,13,15,19,20,21]. This study generated and assessed *N*-nitrosylated derivatives of our previously reported drug-like PC-PLC inhibitors, the 2-morpholinobenzoic acids, in order to synthesize a molecule capable of both inhibiting PC-PLC activity and releasing NO. It was found that the addition of a NO releasing functional group improved anti-proliferative activity across all 13 synthesized analogues of **7a**–**m** when compared to their non-nitrosylated counterparts, **6a**–**m**. The most potent molecule was **7h**, where introduction of the NO donating *N*-nitrosylated moiety significantly increased anti-proliferative activity from 33% inhibition to 96%. This study further substantiates the link between PC-PLC inhibition and exogenous NO release, demonstrating that enhanced anti-proliferative activity is exhibited when both of these functionalities are combined into a single, drug-like molecule. Additionally, this work demonstrates that this relationship can be utilized in order to increase the anti-proliferative potency of previous PC-PLC inhibitors and should be considered when designing future cancer therapeutics that target PC-PLC or other pathways involving phospholipases.

## Figures and Tables

**Table 1:** Relative ^3^H thymidine uptake by MDA-MB-231 cells after treatment with 10 µM of each inhibitor. (green) Compounds that exhibited 60–70% inhibition of cell growth; (blue) compounds that exhibited 70–80% inhibition of cell growth; (red) compounds that exhibited >80% inhibition of cell growth. ** *p* < 0.01, *** *p* < 0.001 when compared to their corresponding non-nitrosylated benzylamine partners by Student’s unpaired *t*-test.

Benzylic Substitution	Relative ^3^H Thymidine Incorporation (% ± s.e.m) at 10 µM in MDA-MB-231 Cells	
	Benzylamines 6 [23]	*N*-Nitrosylated Benzylamines 7
H (**a**)	89.1 ± 1.9	59.0 ± 4.4 **
2-F (**b**)	105.9 ± 0.1	51.4 ± 1.7 ***
3-F (**c**)	85.9 ± 1.6	42.1 ± 4.2 ***
4-F (**d**)	81.4 ± 0.8	44.1 ± 3.0 ***
2-Cl (**e**)	77.1 ± 5.4	30.8 ± 0.5 **
3-Cl (**f**)	79.4 ± 2.7	33.9 ± 0.1 ***
4-Cl (**g**)	77.1 ± 0.9	27.0 ± 0.7 ***
2-Br (**h**)	66.6 ± 3.4	4.1 ± 0.0 ***
3-Br (**i**)	87.4 ± 8.4	24.5 ± 0.7 **
4-Br (**j**)	77.0 ± 2.3	18.8 ± 1.9 ***
2-OMe (**k**)	88.0 ± 5.3	41.6 ± 1.6 **
3-OMe (**l**)	95.7 ± 11.0	71.7 ± 2.5
4-OMe (**m**)	103.0 ± 8.0	25.2 ± 1.5 ***
D609	99.00 ± 2.1	
Positive Control	100 ± 0.9	

**Table 2:** Relative ^3^H thymidine uptake by HCT116 cells after treatment with 10 µM of each inhibitor. (green) Compounds that exhibited 60–70% inhibition of cell growth; (blue) compounds that exhibited 70–80% inhibition of cell growth. * *p* < 0.05, ** *p* < 0.01, *** *p* < 0.001 when compared to their corresponding non-nitrosylated benzylamine partners by Student’s unpaired *t*-test.

Benzylic Substitution	Relative ^3^H Thymidine Incorporation (% ± s.e.m) at 10 µM in HCT-116 Cells	
	Benzylamines 6 [23]	*N*-Nitrosylated Benzylamines 7
H (**a**)	101.3 ± 0.5	75.5 ± 3.4 **
2-F (**b**)	101.1 ± 0.3	75.7 ± 0.4 ***
3-F (**c**)	91.0 ± 4.3	63.2 ± 11.3
4-F (**d**)	89.6 ± 5.5	61.0 ± 3.9 *
2-Cl (**e**)	86.5 ± 1.0	39.7 ± 1.9 ***
3-Cl (**f**)	92.1 ± 0.1	50.1 ± 2.4 ***
4-Cl (**g**)	83.7 ± 1.2	39.5 ± 2.7 ***
2-Br (**h**)	92.0 ± 0.4	24.8 ± 4.4 ***
3-Br (**i**)	95.1 ± 1.8	46.4 ± 3.5 ***
4-Br (**j**)	88.8 ± 0.1	41.3 ± 3.2 ***
2-OMe (**k**)	96.3 ± 0.4	64.9 ± 9.9 *
3-OMe (**l**)	102.6 ± 0.4	84.7 ± 5.0 *
4-OMe (**m**)	100.2 ± 0.3	50.4 ± 7.8 *
D609	98.40 ± 3.2	
Positive Control	100 ± 2.4	

## Supplementary Materials

The following are available online at https://www.mdpi.com/article/10.3390/ijms222111518/s1.
