# Supplementary material for: Incorporation of a Nitric Oxide Donating Motif into Novel PC-PLC Inhibitors Provides Enhanced Anti-Proliferative Activity
Source: Int J Mol Sci. 2021 Oct 26;22(21):11518. doi: 10.3390/ijms222111518 (PMC8583960; doi:10.3390/ijms222111518)
Supplement: Supplementary file 1 [file ijms-22-11518-s001.zip › ijms-1409445-supplementary.pdf]

Supporting Information

Incorporation of a nitric oxide donating motif into novel PC-PLC inhibitors provides enhanced anti-proliferative activity

Shaun W. P. Rees [1], Tayla A. Rees [2,3], Euphemia Leung [3,4], Christopher S. Walker [2,3], David Barker [1,5*], Lisa I. Pilkington [1*].

[1] School of Chemical Sciences, University of Auckland, Auckland, 1010, New Zealand.

[2] School of Biological Science, University of Auckland, Auckland, NZ.

[3] Maurice Wilkins Centre for Molecular Biodiscovery, University of Auckland, Auckland, New Zealand.

[4] Auckland Cancer Society Research Centre, University of Auckland, Grafton, Auckland, 1023, New Zealand.

[5] MacDiarmid Institute for Advanced Materials and Nanotechnology, Wellington, New Zealand.

Table of Contents

General Experimental Details S1

Synthesis of *N*-nitrosylated benzylamines 7a-m: S2

Methyl 5-((benzyl)(nitroso)amino)-2-morpholinobenzoate **7a** S2

Methyl 5-((2'-fluorobenzyl)(nitroso)amino)-2-morpholinobenzoate **7b** S3

Methyl 5-((3'-fluorobenzyl)(nitroso)amino)-2-morpholinobenzoate **7c** S4

Methyl 5-((4'-fluorobenzyl)(nitroso)amino)-2-morpholinobenzoate **7d** S5

Methyl 5-((2'-chlorobenzyl)(nitroso)amino)-2-morpholinobenzoate **7e** S6

Methyl 5-((3'-chlorobenzyl)(nitroso)amino)-2-morpholinobenzoate **7f** S7

Methyl 5-((4'-chlorobenzyl)(nitroso)amino)-2-morpholinobenzoate **7g** S8

Methyl 5-((2'-bromobenzyl)(nitroso)amino)-2-morpholinobenzoate **7h** S9

Methyl 5-((3'-bromobenzyl)(nitroso)amino)-2-morpholinobenzoate **7i** S10

Methyl 5-((4'-bromobenzyl)(nitroso)amino)-2-morpholinobenzoate **7j** S11

Methyl 5-((2'-methoxybenzyl)(nitroso)amino)-2-morpholinobenzoate **7k** ...S12

Methyl 5-((3'-methoxybenzyl)(nitroso)amino)-2-morpholinobenzoate **7l** S13

Methyl 5-((4'-methoxybenzyl)(nitroso)amino)-2-morpholinobenzoate **7m** ..S13

NMR Spectra of *N*-nitrosylated benzylamines 7a-m: S15-27

General experimental details.

NMR spectra were recorded using a Bruker Avance DRX 400 MHz spectrophotometer at ambient temperature. All chemical shifts are reported relative to the solvent peaks of DMSO-d_6 (δ 2.52 for ^{1}H and δ 39.52 for ^{13}C) or $CDCl_3$ (δ 7.26 for ^{1}H and δ 77.16 for ^{13}C). ^{1}H NMR data is reported in the following sequence: position (δ), relative integral, multiplicity (s, singlet; d, doublet; t, triplet; q, quartet; dd, doublet of doublets; m, multiplet; br s, broad singlet; br d, broad doublet), coupling constant (*J*, Hz), and proton assignment. ^{13}C NMR data was reported in the following sequence: position (δ), multiplicity (d, doublet), coupling constant (*J*, Hz), and the carbon assignment. NMR assignments were made using a combination of ^{1}H NMR, ^{13}C NMR, HSQC and HMBC experiments. High-resolution mass spectroscopy (HRMS) was carried out using electrospray ionisation (ESI) on a MicroTOF-Q mass spectrometer. All reactions were carried out using dried AR grade solvents under a nitrogen atmosphere, all chemical reagents were purchased from AK scientific (Union City, CA, USA) and used as purchased.

Thin-layer chromatography (TLC) was carried out on Merck silica gel F354 aluminium plates pre-coated with silica and flash chromatography was conducted with Silica Gel 60 (40–63 μm, 230-430 mesh ASTM) using solvent systems specified in the experimental procedures. Melting points were measured using a Reicher-Kofler block, able to measure melting points up to 230 °C; melting points reported in this study are uncorrected. IR spectra were recorded using a Perkin-Elmer Spectrum 1000 series Fourier Transform Infra-Red ATR spectrometer. High-resolution mass spectroscopy (HRMS) was carried out using electrospray ionisation (ESI) on a MicroTOF-Q mass spectrometer.

Synthesis of *N*-nitrosylated benzylamines 7a-m

Methyl 5-((benzyl)(nitroso)amino)-2-morpholinobenzoate 7a

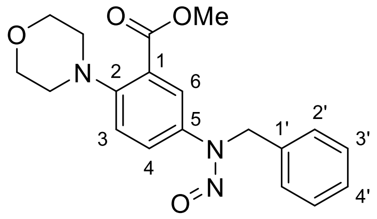

The reaction was carried out following General Procedure 3.1.2, methyl 5-((benzyl)amino)-2-morpholinobenzoate **6a** (0.075 g, 0.23 mmol), $NaNO_2$ (0.017 g. 0.24 mmol) and *para*-toluenesulfonic acid monohydrate (0.043g, 0.24 mmol) in CH_2Cl_2 (5 mL) for 19 h. The crude product was purified using flash chromatography (4:1 petroleum ether, ethyl acetate) to give the *title product* **7a** (0.069 g, 84%) as a yellow solid. M.p.: 79 - 81 °C. Rf: 0.34 (2:1 petroleum ether, ethyl acetate). IR: ν_{max} (film)/cm^{-1}; 2953 (CH aromatic), 2872 (CH alkane), 1720 (C=O ester), 1451 (N=O), 1211 and 1129 (C-O ether). δ_H (400 MHz; $(CD_3)_2SO$) 2.98 (4H, t, *J* = 4.5 Hz, N(C*H*$_2$CH$_2$)$_2$O), 3.70 (4H, t, *J* = 4.5 Hz, N(CH$_2$C*H*$_2$)$_2$O), 3.81 (3H, s, COOCH$_3$), 5.32 (2H, s, CH$_2$), 7.06 (2H, d, *J* = 7.5 Hz, H-2′), 7.19 – 7.25 (2H, m, H-3, H-4'), 7.29 (2H, t, *J* = 7.5 Hz, H-3′), 7.70 (1H, dd, *J* = 9.0, 3.0 Hz, H-4) and 7.85 (1H, d, *J* = 3.0 Hz, H-6). δ_C (100 MHz; $(CD_3)_2SO$) 46.2 (CH$_2$), 51.9 (N(CH$_2$CH$_2$)$_2$O), 52.2 (COOCH$_3$), 66.2 (N(CH$_2$CH$_2$)$_2$O), 119.8 (C-3), 122.5 (C-6), 124.1 (C-1), 124.2 (C-4), 127.1 (C-2'), 127.4 (C-4'), 128.7 (C-3'), 134.3 (C-5), 134.5 (C-1'), 150.5 (C-2) and 167.1 (C=O). *m*/*z* (ESI+): 378 (MNa$^+$, 50%), 348 (100), 326 (30). HRMS (ESI+): Found (MNa+): 378.1429, $C_{19}H_{21}N_3NaO_4$ requires 378.1424.

Methyl 5-((2-fluorobenzyl)(nitroso)amino)-2-morpholinobenzoate 7b

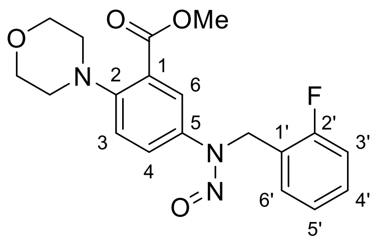

The reaction was carried out following General Procedure 3.1.2, using methyl 5-((2-fluorobenzyl)amino)-2-morpholinobenzoate **6b** (0.075 g, 0.22 mmol), $NaNO_2$ (0.016 g. 0.23 mmol) and *para*-toluenesulfonic acid monohydrate (0.044g, 0.23 mmol) in CH_2Cl_2 (5 mL) for 26 h. The crude product was purified using flash chromatography (4:1 petroleum ether, ethyl acetate) to give the *title product* **7b** (0.060 g, 74%) as a brown solid. M.p.: 89 - 90 °C. Rf: 0.43 (2:1 petroleum ether, ethyl acetate). IR: ν_{max} (film)/cm^{-1}; 2972 (CH aromatic), 2860 (CH alkane), 1721 (C=O ester), 1453 (N=O), 1229 and 1129 (C-O ether). δ_H (400 MHz; $(CD_3)_2CO$) 3.05 (4H, t, J = 4.5 Hz, N(CH_2CH$_2$)$_2$O), 3.76 (4H, t, J = 4.5 Hz, N(CH$_2$CH_2)$_2$O), 3.86 (3H, s, $COOCH_3$), 5.35 (2H, s, CH_2), 7.05 (1H, t, J = 7.5 Hz, H-6′), 7.08 – 7.14 (2H, m, H-3′ and H-5′), 7.23 (1H, d, J = 9.0 Hz, H-3), 7.30 (1H, q, J = 7.5 Hz, H-4′), 7.69 (1H, dd, J = 9.0, 3.0 Hz, H-4) and 7.92 (1H, d, J = 3.0 Hz, H-6). δ_C (100 MHz; $(CD_3)_2CO$) 41.7 (CH_2), 52.5 ($COOCH_3$), 53.4 ($N(CH_2CH_2)_2O$), 67.5 ($N(CH_2CH_2)_2O$), 116.3 (d, $^2J_{F/C}$ = 21.7 Hz, C-3′), 120.8 (C-3), 122.5 (d, $^2J_{F/C}$ = 14.7 Hz, C-1′), 124.1 (C-6), 125.3 (C-4), 125.4 (d, $^4J_{F/C}$ = 3.1 Hz, C-5′), 126.0 (C-1), 130.3 (d, $^3J_{F/C}$ = 3.7 Hz, C-4′), 130.5 (d, $^3J_{F/C}$ = 8.4 Hz, C-6′), 135.9 (C-5), 152.2 (C-2), 161.3 (d, $^1J_{F/C}$ = 245.5 Hz, C-2′) and 167.9 (C=O). *m/z* (ESI+): 396 (MNa^+, 90%), 366 (100), 344 (50). HRMS (ESI+): Found (MNa+): 396.1327, $C_{19}H_{21}N_3NaO_4$ requires 396.1330.

Methyl 5-((3-fluorobenzyl)(nitroso)amino)-2-morpholinobenzoate 7c

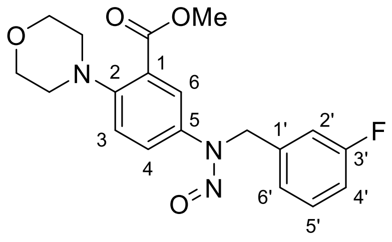

The reaction was carried out following General Procedure 3.1.2, using methyl 5-((3-fluorobenzyl)amino)-2-morpholinobenzoate **6c** (0.075 g, 0.22 mmol), $NaNO_2$ (0.016 g. 0.23 mmol) and *para*-toluenesulfonic acid monohydrate (0.044g, 0.23 mmol) in CH_2Cl_2 (5 mL) for 22 h. The crude product was purified using flash chromatography (4:1 petroleum ether, ethyl acetate) to give the *title product* **7c** (0.076 g, 94%) as a brown solid. M.p.: 83 - 85 °C. Rf: 0.43 (2:1 petroleum ether, ethyl acetate). IR: ν_{max} (film)/cm^{-1}; 2951 (CH aromatic), 2849 (CH alkane), 1724 (C=O ester), 1453 (N=O), 1229 and 1113 (C-O ether). δ_H (400 MHz; $CDCl_3$) 3.07 (4H, t, *J* = 4.5 Hz, N(C*H*$_2$CH$_2$)$_2$O), 3.86 (4H, t, *J* = 4.5 Hz, N(CH$_2$C*H*$_2$)$_2$O), 3.89 (3H, s, $COOCH_3$), 5.20 (2H, s, CH_2), 6.79 (1H, dt, *J* = 9.5, 2.0 Hz, H-2′), 6.87 (1H, dd, *J* = 8.5, 1.0 Hz, H-6'), 6.95 (1H, td, *J* = 8.5, 2.5 Hz, H-4'), 7.09 (1H, d, *J* = 9.0 Hz, H-3), 7.23 – 7.29 (1H, m, H-5′), 7.55 (1H, dd, *J* = 9.0, 3.0 Hz, H-4) and 7.93 (1H, d, *J* = 3.0 Hz, H-6). δ_C (100 MHz; $CDCl_3$) 46.9 (CH_2), 52.5 ($COOCH_3$), 52.9 (N(*C*H$_2$CH$_2$)$_2$O), 67.1 (N(CH$_2$*C*H$_2$)$_2$O), 114.5 (d, $^2J_{F/C}$ = 22.4 Hz, C-2′), 115.0 (d, $^2J_{F/C}$ = 21.0 Hz, C-4′), 120.0 (C-3), 123.0 (d, $^4J_{F/C}$ = 2.8 Hz, C-6′), 123.3 (C-6), 124.2 (C-4), 124.7 (C-1), 130.6 (d, $^3J_{F/C}$ = 8.3 Hz, C-5'), 135.4 (C-5), 136.6 (d, $^3J_{F/C}$ = 7.3 Hz, C-1′), 151.7 (C-2), 163.1 (d, $^1J_{F/C}$ = 247.1 Hz, C-3′) and 167.1 (C=O). *m/z* (ESI+): 396 (MNa^+, 100%), 366 (90), 344 (45). HRMS (ESI+): Found (MNa+): 396.1334, $C_{19}H_{21}N_3NaO_4$ requires 396.1330.

Methyl 5-((4-fluorobenzyl)(nitroso)amino)-2-morpholinobenzoate 7d

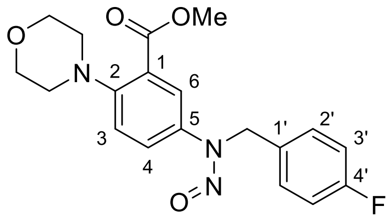

The reaction was carried out following General Procedure 3.1.2, using methyl 5-((4-fluorobenzyl)amino)-2-morpholinobenzoate **6d** (0.075 g, 0.22 mmol), $NaNO_2$ (0.016 g. 0.23 mmol) and *para*-toluenesulfonic acid monohydrate (0.044g, 0.23 mmol) in CH_2Cl_2 (5 mL) for 20 h. The crude product was purified using flash chromatography (4:1 petroleum ether, ethyl acetate) to give the *title product* **7d** (0.067 g, 82%) as a yellow solid. M.p.: 73 - 75 °C. Rf: 0.46 (2:1 petroleum ether, ethyl acetate). IR: ν_{max} (film)/cm^{-1}; 2956 (CH aromatic), 2863 (CH alkane), 1724 (C=O ester), 1457 (N=O), 1221 and 1117 (C-O ether). δ_H (400 MHz; $(CD_3)_2CO$) 3.04 (4H, t, J = 4.5 Hz, N(CH_2CH$_2$)$_2$O), 3.76 (4H, t, J = 4.5 Hz, N(CH$_2$CH_2)$_2$O), 3.86 (3H, s, $COOCH_3$), 5.32 (2H, s, CH_2), 7.06 (2H, t, J = 8.0 Hz, H-3′), 7.18 (2H, t, J = 8.0 Hz, H-2'), 7.23 (1H, d, J = 9.0 Hz, H-3), 7.68 (1H, dd, J = 9.0, 3.0 Hz, H-4) and 7.90 (1H, d, J = 3.0 Hz, H-6). δ_C (100 MHz; $(CD_3)_2CO$) 46.6 (CH_2), 52.5 ($COOCH_3$), 53.4 (N(CH_2CH$_2$)$_2$O), 67.5 (N(CH$_2$$CH_2$)$_2$O), 116.3 (d, $^2J_{F/C}$ = 22.1 Hz, C-3′), 120.8 (C-3), 123.9 (C-6), 125.1 (C-4), 126.1 (C-1), 130.4 (d, $^3J_{F/C}$ = 8.5 Hz, C-2'), 131.9 (d, $^4J_{F/C}$ = 3.5 Hz, C-1′), 136.0 (C-5), 152.1 (C-2), 162.9 (d, $^1J_{F/C}$ = 244.2 Hz, C-4′) and 168.0 (C=O). *m/z* (ESI+): 396 (MNa^+, 90%), 366 (100), 344 (50). HRMS (ESI+): Found (MNa+): 396.1331, $C_{19}H_{21}N_3NaO_4$ requires 396.1330.

Methyl 5-((2-chlorobenzyl)(nitroso)amino)-2-morpholinobenzoate 7e

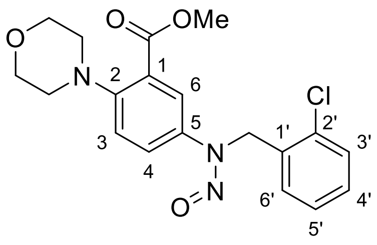

The reaction was carried out following General Procedure 3.1.2, using methyl 5-((2-chlorobenzyl)amino)-2-morpholinobenzoate **6e** (0.075 g, 0.21 mmol), $NaNO_2$ (0.016 g. 0.22 mmol) and *para*-toluenesulfonic acid monohydrate (0.044g, 0.22 mmol) in CH_2Cl_2 (5 mL) for 3 d. The crude product was purified using flash chromatography (4:1 petroleum ether, ethyl acetate) to give the *title product* **7e** (0.061 g, 75%) as an orange solid. M.p.: 72 - 74 °C. Rf: 0.50 (2:1 petroleum ether, ethyl acetate). IR: ν_{max} (film)/cm^{-1}; 2957 (CH aromatic), 2860 (CH alkane), 1726 (C=O ester), 1463 (N=O), 1231 and 1112 (C-O ether). δ_H (400 MHz; $CDCl_3$) 3.06 (4H, t, J = 4.5 Hz, N(C$H_2$$CH_2$)$_2$O), 3.86 (4H, t, J = 4.5 Hz, N(CH_2CH_2)$_2$O), 3.89 (3H, s, $COOCH_3$), 5.30 (2H, s, CH_2), 6.81 (1H, dd, J = 7.5, 1.5 Hz, H-6′), 7.08 (1H, d, J = 9.0 Hz, H-3), 7.14 (1H, td, J = 7.5, 1.5 Hz, H-5'), 7.20 (1H, td, J = 7.5, 2.0 Hz, H-4'), 7.37 (1H, dd, J = 8.0, 1.5 Hz, H-3'), 7.54 (1H, dd, J = 9.0, 3.0 Hz, H-4) and 7.95 (1H, d, J = 3.0 Hz, H-6). δ_C (100 MHz; $CDCl_3$) 45.2 (CH_2), 52.5 ($COOCH_3$), 52.9 (N(CH_2CH_2)$_2$O), 67.1 (N(CH_2CH_2)$_2$O), 119.9 (C-3), 123.1 (C-6), 123.9 (C-4), 124.8 (C-1), 127.4 (C-5'), 128.1 (C-6'), 129.2 (C-4'), 129.9 (C-3'), 131.3 (C-1'), 132.9 (C-2'), 135.4 (C-5), 151.6 (C-2) and 167.2 (C=O). *m/z* (ESI+): 414 (^{37}ClMNa$^+$, 30%), 412 (^{35}ClMNa$^+$, 90), 384 (33), 382 (100). HRMS (ESI+): Found (MNa$^+$): 414.0997, $C_{19}H_{20}{}^{37}ClN_3NaO_4$ requires 414.1012. Found (MNa$^+$): 412.1021, $C_{19}H_{20}{}^{35}ClN_3NaO_4$ requires 412.1035.

Methyl 5-((3-chlorobenzyl)(nitroso)amino)-2-morpholinobenzoate 7f

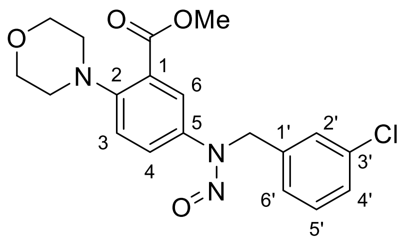

The reaction was carried out following General Procedure 3.1.2, using methyl 5-((3-chlorobenzyl)amino)-2-morpholinobenzoate **6f** (0.075 g, 0.21 mmol), $NaNO_2$ (0.016 g. 0.22 mmol) and *para*-toluenesulfonic acid monohydrate (0.044g, 0.22 mmol) in CH_2Cl_2 (5 mL) for 2 d. The crude product was purified using flash chromatography (4:1 petroleum ether, ethyl acetate) to give the *title product* **6f** (0.051 g, 63%) as a red solid. M.p.: 84 - 86 °C. Rf: 0.37 (2:1 petroleum ether, ethyl acetate). IR: ν_{max} (film)/cm^{-1}; 2971 (CH aromatic), 2821 (CH alkane), 1728 (C=O ester), 1433 (N=O), 1224 and 1111 (C-O ether). δ_H (400 MHz; $(CD_3)_2SO$) 2.98 (4H, t, *J* = 4.5 Hz, N(C*H*$_2$CH$_2$)$_2$O), 3.70 (4H, t, *J* = 4.5 Hz, N(CH$_2$C*H*$_2$)$_2$O), 3.82 (3H, s, COOCH$_3$), 5.33 (2H, s, CH$_2$), 7.00 (1H, t, *J* = 7.5 Hz, H-6′), 7.17 (1H, s, H-2'), 7.21 (1H, d, *J* = 9.0 Hz, H-3), 7.30 – 7.33 (2H, m, H-4' and H-5'), 7.70 (1H, dd, *J* = 9.0, 3.0 Hz, H-4) and 7.86 (1H, d, *J* = 3.0 Hz, H-6). δ_C (100 MHz; $(CD_3)_2SO$) 45.8 (CH$_2$), 51.9 (N(CH$_2$CH$_2$)$_2$O), 52.3 (COOCH$_3$), 66.2 (N(CH$_2$CH$_2$)$_2$O), 119.8 (C-3), 122.4 (C-6), 124.0 (C-1), 124.2 (C-4), 125.6 (C-6'), 127.1 (C-2'), 127.5 (C-4'), 130.6 (C-5'), 133.2 (C-3'), 134.1 (C-5), 137.0 (C-1'), 150.5 (C-2) and 167.0 (C=O). *m/z* (ESI+): 414 (^{37}ClMNa$^+$, 33%), 412 (^{35}ClMNa$^+$, 100), 384 (30), 382 (90). HRMS (ESI+): Found (MNa$^+$): 414.0985, $C_{19}H_{20}{}^{37}ClN_3NaO_4$ requires 414.1012. Found (MNa$^+$): 412.1024, $C_{19}H_{20}{}^{35}ClN_3NaO_4$ requires 412.1035.

Methyl 5-((4-chlorobenzyl)(nitroso)amino)-2-morpholinobenzoate 7g

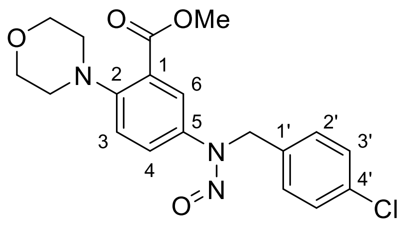

The reaction was carried out following General Procedure 3.1.2, using methyl 5-((4-chlorobenzyl)amino)-2-morpholinobenzoate **6g** (0.075 g, 0.21 mmol), $NaNO_2$ (0.016 g. 0.22 mmol) and *para*-toluenesulfonic acid monohydrate (0.044g, 0.22 mmol) in CH_2Cl_2 (5 mL) for 18 h. The crude product was purified using flash chromatography (4:1 petroleum ether, ethyl acetate) to give the *title product* **7g** (0.066 g, 81%) as a light brown solid. M.p.: 86 - 88 °C. Rf: 0.35 (2:1 petroleum ether, ethyl acetate). IR: ν_{max} (film)/cm^{-1}; 2962 (CH aromatic), 2821 (CH alkane), 1708 (C=O ester), 1446 (N=O), 1265 and 1114 (C-O ether). δ_H (400 MHz; $(CD_3)_2SO$) 2.98 (4H, t, *J* = 4.5 Hz, N(C*H*$_2$CH$_2$)$_2$O), 3.70 (4H, t, *J* = 4.5 Hz, N(CH$_2$C*H*$_2$)$_2$O), 3.82 (3H, s, COOCH$_3$), 5.31 (2H, s, CH$_2$), 7.10 (2H, d, *J* = 8.0 Hz, H-2'), 7.20 (1H, d, *J* = 9.0 Hz, H-3), 7.35 (2H, d, *J* = 8.0 Hz, H-3'), 7.69 (1H, dd, *J* = 9.0, 3.0 Hz, H-4) and 7.84 (1H, d, *J* = 3.0 Hz, H-6). δ_C (100 MHz; $(CD_3)_2SO$) 45.7 (CH$_2$), 51.9 (N(CH$_2$CH$_2$)$_2$O), 52.3 (COOCH$_3$), 66.2 (N(CH$_2$CH$_2$)$_2$O), 119.8 (C-3), 122.5 (C-6), 124.2 (C-1), 124.3 (C-4), 128.7 (C-3'), 129.1 (C-2'), 132.0 (C-4'), 133.5 (C-1'), 134.1 (C-5), 150.3 (C-2) and 167.0 (C=O). *m/z* (ESI+): 414 (^{37}ClMNa$^+$, 33%), 412 (^{35}ClMNa$^+$, 100), 398 (90), 396 (30). HRMS (ESI+): Found (MNa$^+$): 414.1026, $C_{19}H_{20}{}^{37}ClN_3NaO_4$ requires 414.1012. Found (MNa$^+$): 412.1021, $C_{19}H_{20}{}^{35}ClN_3NaO_4$ requires 412.1035.

Methyl 5-((2-bromobenzyl)(nitroso)amino)-2-morpholinobenzoate 7h

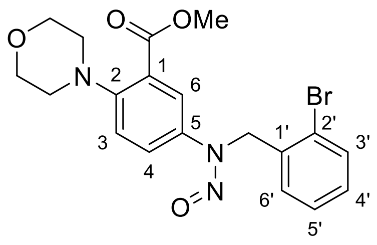

The reaction was carried out following General Procedure 3.1.2, using methyl 5-((2-bromobenzyl)amino)-2-morpholinobenzoate **6h** (0.075 g, 0.19 mmol), $NaNO_2$ (0.016 g. 0.20 mmol) and *para*-toluenesulfonic acid monohydrate (0.044g, 0.20 mmol) in CH_2Cl_2 (5 mL) for 18 h. The crude product was purified using flash chromatography (4:1 petroleum ether, ethyl acetate) to give the *title product* **7h** (0.052 g, 65%) as a yellow solid. M.p.: 103 - 105 °C. Rf: 0.43 (2:1 petroleum ether, ethyl acetate). IR: ν_{max} (film)/cm^{-1}; 2948 (CH aromatic), 2849 (CH alkane), 1721 (C=O ester), 1442 (N=O), 1210 and 1113 (C-O ether). δ_H (400 MHz; $CDCl_3$) 3.06 (4H, t, *J* = 4.5 Hz, $N(CH_2CH_2)_2O$), 3.86 (4H, t, *J* = 4.5 Hz, $N(CH_2CH_2)_2O$), 3.89 (3H, s, $COOCH_3$), 5.27 (2H, s, CH_2), 6.77 (1H, dd, *J* = 7.5, 1.5 Hz, H-6′), 7.08 (1H, d, *J* = 9.0 Hz, H-3), 7.12 (1H, td, *J* = 7.5, 2.0 Hz, H-4'), 7.19 (1H, td, *J* = 7.5, 1.5 Hz, H-5'), 7.53 (1H, dd, *J* = 9.0, 3.0 Hz, H-4), 7.57 (1H, dd, *J* = 7.5, 1.5 Hz, H-3'), and 7.96 (1H, d, *J* = 3.0 Hz, H-6). δ_C (100 MHz; $CDCl_3$) 47.8 (CH_2), 52.5 ($COOCH_3$), 52.9 ($N(CH_2CH_2)_2O$), 67.1 ($N(CH_2CH_2)_2O$), 119.9 (C-3), 122.7 (C-2'), 123.0 (C-6), 123.8 (C-4), 124.8 (C-1), 128.0 (C-5' and C-6'), 129.4 (C-4'), 132.8 (C-1'), 133.2 (C-3'), 135.4 (C-5), 151.6 (C-2) and 167.2 (C=O). *m/z* (ESI+): 458 ($^{81}BrMNa^+$, 100%), 456 ($^{79}BrMNa^+$, 100), 428 (50), 426 (50). HRMS (ESI+): Found (MNa^+): 458.0502, $C_{19}H_{20}{}^{81}BrN_3NaO_4$ requires 458.0511. Found (MNa^+): 456.0526, $C_{19}H_{20}{}^{79}BrN_3NaO_4$ requires 456.0529.

Methyl 5-((3-bromobenzyl)(nitroso)amino)-2-morpholinobenzoate 7i

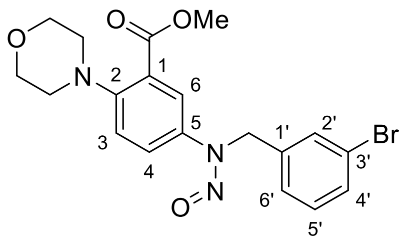

The reaction was carried out following General Procedure 3.1.2, using methyl 5-((3-bromobenzyl)amino)-2-morpholinobenzoate **6i** (0.075 g, 0.19 mmol), $NaNO_2$ (0.016 g. 0.20 mmol) and *para*-toluenesulfonic acid monohydrate (0.044g, 0.20 mmol) in CH_2Cl_2 (5 mL) for 20 h. The crude product was purified using flash chromatography (4:1 petroleum ether, ethyl acetate) to give the *title product* **7i** (0.075 g, 94%) as a red/orange oil. Rf: 0.40 (2:1 petroleum ether, ethyl acetate). IR: ν_{max} (film)/cm^{-1}; 2948 (CH aromatic), 2863 (CH alkane), 1721 (C=O ester), 1432 (N=O), 1229 and 1113 (C-O ether). δ_H (400 MHz; $CDCl_3$) 3.08 (4H, t, *J* = 4.5 Hz, N(C*H*$_2$CH$_2$)$_2$O), 3.87 (4H, t, *J* = 4.5 Hz, N(CH$_2$C*H*$_2$)$_2$O), 3.90 (3H, s, $COOCH_3$), 5.17 (2H, s, CH_2), 7.00 (1H, dt, *J* = 8.5, 1.0 Hz, H-6'), 7.09 (1H, d, *J* = 9.0 Hz, H-3), 7.16 (1H, t, *J* = 8.5 Hz, H-5'), 7.25 (1H, t, *J* = 1.5 Hz, H-2'), 7.38 – 7.40 (1H, m, H-4'), 7.55 (1H, dd, *J* = 9.0, 3.0 Hz, H-4) and 7.93 (1H, d, *J* = 3.0 Hz, H-6). δ_C (100 MHz; $CDCl_3$) 46.8 (CH_2), 52.5 ($COOCH_3$), 52.8 (N(CH_2CH_2)$_2$O), 67.1 (N(CH_2CH_2)$_2$O), 120.0 (C-3), 123.1 (C-3'), 123.3 (C-6), 124.2 (C-4), 124.6 (C-1), 126.0 (C-6'), 130.5 (C-2'), 130.6 (C-5'), 131.1 (C-4'), 135.3 (C-5), 136.5 (C-1'), 151.7 (C-2) and 167.1 (C=O). *m/z* (ESI+): 458 (^{81}BrMNa$^+$, 100%), 456 (^{79}BrMNa$^+$, 100), 428 (50), 426 (50). HRMS (ESI+): Found (MNa$^+$): 458.0495, $C_{19}H_{20}{}^{81}BrN_3NaO_4$ requires 458.0511. Found (MNa$^+$): 456.0518, $C_{19}H_{20}{}^{79}BrN_3NaO_4$ requires 456.0529.

Methyl 5-((4-bromobenzyl)(nitroso)amino)-2-morpholinobenzoate 7j

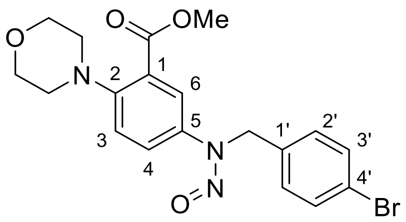

The reaction was carried out following General Procedure 3.1.2, using methyl 5-((4-bromobenzyl)amino)-2-morpholinobenzoate **6j** (0.075 g, 0.19 mmol), $NaNO_2$ (0.016 g. 0.20 mmol) and *para*-toluenesulfonic acid monohydrate (0.044g, 0.20 mmol) in CH_2Cl_2 (5 mL) for 25 h. The crude product was purified using flash chromatography (4:1 petroleum ether, ethyl acetate) to give the *title product* **7j** (0.068 g, 85%) as a reddish brown solid. M.p.: 90 - 92 °C. Rf: 0.41 (2:1 petroleum ether, ethyl acetate). IR: ν_{max} (film)/cm^{-1}; 2963 (CH aromatic), 2868 (CH alkane), 1709 (C=O ester), 1444 (N=O), 1227 and 1114 (C-O ether). δ_H (400 MHz; $(CD_3)_2SO$) 2.98 (4H, t, J = 4.5 Hz, $N(CH_2CH_2)_2O$), 3.70 (4H, t, J = 4.5 Hz, $N(CH_2CH_2)_2O$), 3.82 (3H, s, $COOCH_3$), 5.29 (2H, s, CH_2), 7.02 (2H, d, J = 8.5 Hz, H-2'), 7.19 (1H, d, J = 9.0 Hz, H-3), 7.49 (2H, d, J = 8.5 Hz, H-3'), 7.69 (1H, dd, J = 9.0, 3.0 Hz, H-4) and 7.84 (1H, d, J = 3.0 Hz, H-6). δ_C (100 MHz; $(CD_3)_2SO$) 45.8 (CH_2), 51.9 ($N(CH_2CH_2)_2O$), 52.2 ($COOCH_3$), 66.2 ($N(CH_2CH_2)_2O$), 119.8 (C-3), 120.5 (C-4'), 122.5 (C-6), 124.2 (C-1), 124.3 (C-4), 129.4 (C-2'), 131.6 (C-3'), 133.9 (C-1'), 134.1 (C-5), 150.5 (C-2) and 167.0 (C=O). *m/z* (ESI+): 458 ($^{81}BrMNa^+$, 100%), 456 ($^{79}BrMNa^+$, 100), 428 (50), 426 (50). HRMS (ESI+): Found (MNa^+): 458.0509, $C_{19}H_{20}{}^{81}BrN_3NaO_4$ requires 458.0511. Found (MNa^+): 456.0526, $C_{19}H_{20}{}^{79}BrN_3NaO_4$ requires 456.0529.

Methyl 5-((2-methoxybenzyl)(nitroso)amino)-2-morpholinobenzoate 7k

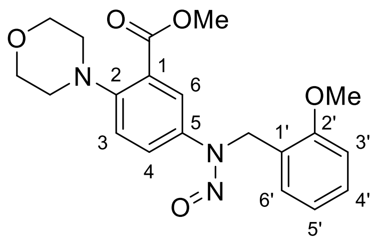

The reaction was carried out following General Procedure 3.1.2, methyl 5-((2-methoxybenzyl)amino)-2-morpholinobenzoate **6k** (0.075 g, 0.21 mmol), $NaNO_2$ (0.016 g. 0.22 mmol) and *para*-toluenesulfonic acid monohydrate (0.042g, 0.22 mmol) in CH_2Cl_2 (5 mL) for 22 h. The crude product was purified using flash chromatography (4:1 petroleum ether, ethyl acetate) to give the *title product* **7k** (0.067 g, 82%) as a yellow/brown solid. M.p.: 75 – 77 °C. Rf: 0.23 (3:1 petroleum ether, ethyl acetate). IR: ν_{max} (film)/cm^{-1}; 2963 (CH aromatic), 2843 (CH alkane), 1708 (C=O ester), 1435 (N=O), 1210 and 1113 (C-O ether). δ_H (400 MHz; $(CD_3)_2SO$) 2.97 (4H, t, *J* = 4.5 Hz, N(C*H*$_2$CH$_2$)$_2$O), 3.69 (4H, t, *J* = 4.5 Hz, N(CH$_2$C*H*$_2$)$_2$O), 3.75 (3H, s, 2'-OCH$_3$), 3.81 (3H, s, COOCH$_3$), 5.18 (2H, s, CH$_2$), 6.78 – 6.84 (2H, m, H-5' and H-6'), 6.97 (1H, d, *J* = 8.0 Hz, H-3'), 7.18 (1H, d, *J* = 9.0 Hz, H-3), 7.20 – 7.25 (1H, m, H-4'), 7.65 (1H, dd, *J* = 9.0, 3.0 Hz, H-4) and 7.81 (1H, d, *J* = 3.0 Hz, H-6). δ_C (100 MHz; $(CD_3)_2SO$) 42.4 (CH$_2$), 51.9 (N(*C*H$_2$CH$_2$)$_2$O), 52.2 (COOCH$_3$), 55.4 (2′-OCH$_3$), 66.2 (N(CH$_2$CH$_2$)$_2$O), 110.9 (C-3′), 119.7 (C-3), 120.3 (C-5'), 121.6 (C-1'), 122.7 (C-6), 123.9 (C-1), 124.5 (C-4), 127.9 (C-6'), 128.9 (C-4'), 134.5 (C-5), 150.5 (C-2), 156.6 (C-2') and 167.0 (C=O). *m/z* (ESI+): 408 (MNa$^+$, 40%), 378 (100). HRMS (ESI+): Found (MNa$^+$): 408.1520, $C_{20}H_{23}N_3NaO_5$ requires 408.1530.

Methyl 5-((3-methoxybenzyl)(nitroso)amino)-2-morpholinobenzoate 7l

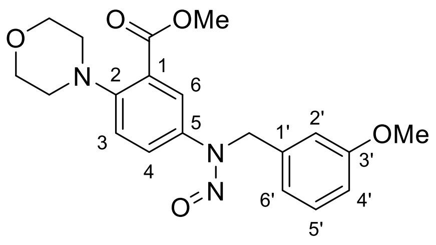

The reaction was carried out following General Procedure 3.1.2, methyl 5-((3-methoxybenzyl)amino)-2-morpholinobenzoate **6l** (0.075 g, 0.21 mmol), $NaNO_2$ (0.016 g. 0.22 mmol) and *para*-toluenesulfonic acid monohydrate (0.042g, 0.22 mmol) in CH_2Cl_2 (5 mL) for 17 h. The crude product was purified using flash chromatography (4:1 petroleum ether, ethyl acetate) to give the *title product* **7l** (0.064 g, 78%) as a red/orange oil. Rf: 0.34 (2:1 petroleum ether, ethyl acetate). IR: ν_{max} (film)/cm^{-1}; 2963 (CH aromatic), 2840 (CH alkane), 1720 (C=O ester), 1435 (N=O), 1228 and 1114 (C-O ether). δ_H (400 MHz; $CDCl_3$) 3.07 (4H, t, *J* = 4.5 Hz, N(C$H_2$$CH_2$)$_2$O), 3.75 (3H, s, 3'-$OCH_3$), 3.86 (4H, t, *J* = 4.5 Hz, N(CH_2CH_2)$_2$O), 3.89 (3H, s, $COOCH_3$), 5.19 (2H, s, CH_2), 6.61 (1H, t, *J* = 2.0 Hz, H-2'), 6.67 (1H, d, *J* = 8.0 Hz, H-6′), 6.77 (1H, dd, *J* = 8.0, 2.0 Hz, H-4'), 7.07 (1H, d, *J* = 9.0 Hz, H-3), 7.20 (1H, t, *J* = 8.0 Hz, H-5'), 7.55 (1H, dd, *J* = 9.0, 3.0 Hz, H-4) and 7.97 (1H, d, *J* = 3.0 Hz, H-6). δ_C (100 MHz; $CDCl_3$) 47.2 (CH_2), 52.9 (N(C$H_2$$CH_2$)$_2$O), 52.5 ($COOCH_3$), 55.4 (3′-$OCH_3$), 67.1 (N($CH_2$$CH_2$)$_2$O), 113.1 (C-2'), 113.3 (C-4'), 119.6 (C-6'), 120.0 (C-3), 123.3 (C-6), 124.1 (C-4), 124.8 (C-1), 130.1 (C-5'), 135.8 (C-1'), 135.9 (C-5), 151.3 (C-2), 160.1 (C-3') and 167.2 (C=O). *m*/*z* (ESI+): 408 (MNa^+, 60%), 378 (100). HRMS (ESI+): Found (MNa^+): 408.1527, $C_{20}H_{23}N_3NaO_5$ requires 408.1530.

Methyl 5-((4-methoxybenzyl)(nitroso)amino)-2-morpholinobenzoate 7m

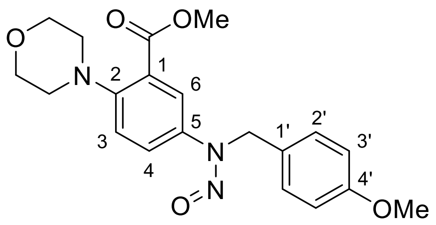

The reaction was carried out following General Procedure 3.1.2, methyl 5-((4-methoxybenzyl)amino)-2-morpholinobenzoate **6m** (0.075 g, 0.21 mmol), $NaNO_2$ (0.016 g. 0.22 mmol) and *para*-toluenesulfonic acid monohydrate (0.042g, 0.22 mmol) in CH_2Cl_2 (5 mL) for 17 h. The crude product was purified using flash chromatography (4:1 petroleum ether, ethyl acetate) to give the *title product* **7m** (0.055 g, 73%) as a red solid M.p.: 103 – 105 °C. Rf: 0.31 (2:1 petroleum ether, ethyl acetate). IR: ν_{max} (film)/cm^{-1}; 2967 (CH aromatic), 2840 (CH alkane), 1706 (C=O ester), 1446 (N=O), 1249 and 1113 (C-O ether). δ_H (400 MHz; $(CD_3)_2SO$) 2.97 (4H, t, *J* = 4.5 Hz, N(C*H*$_2$CH$_2$)$_2$O), 3.67 (4H, t, *J* = 4.5 Hz, N(CH$_2$C*H*$_2$)$_2$O), 3.68 (3H, s, 4'-OCH$_3$), 3.81 (3H, s, COOCH$_3$), 5.24 (2H, s, CH$_2$), 6.83 (2H, d, *J* = 9.0 Hz, H-3'), 7.00 (2H, d, *J* = 9.0 Hz, H-2'), 7.19 (1H, d, *J* = 9.0 Hz, H-3), 7.68 (1H, dd, *J* = 9.0, 3.0 Hz, H-4) and 7.83 (1H, d, *J* = 3.0 Hz, H-6). δ_C (100 MHz; $(CD_3)_2SO$) 45.5 (CH$_2$), 51.9 (N(*C*H$_2$CH$_2$)$_2$O), 52.2 (COO*C*H$_3$), 55.0 (4′-OCH$_3$), 66.1 (N(CH$_2$*C*H$_2$)$_2$O), 114.1 (C-3'), 119.7 (C-3), 122.6 (C-6), 124.1 (C-1), 124.4 (C-4), 126.3 (C-1'), 128.7 (C-2'), 134.2 (C-5), 150.4 (C-2), 158.5 (C-4') and 167.0 (C=O). *m/z* (ESI+): 408 (MNa$^+$, 50%), 378 (90), 227 (100). HRMS (ESI+): Found (MNa$^+$): 408.1528, $C_{20}H_{23}N_3NaO_5$ requires 408.1530.

NMR Spectra of Compounds 7a-m:

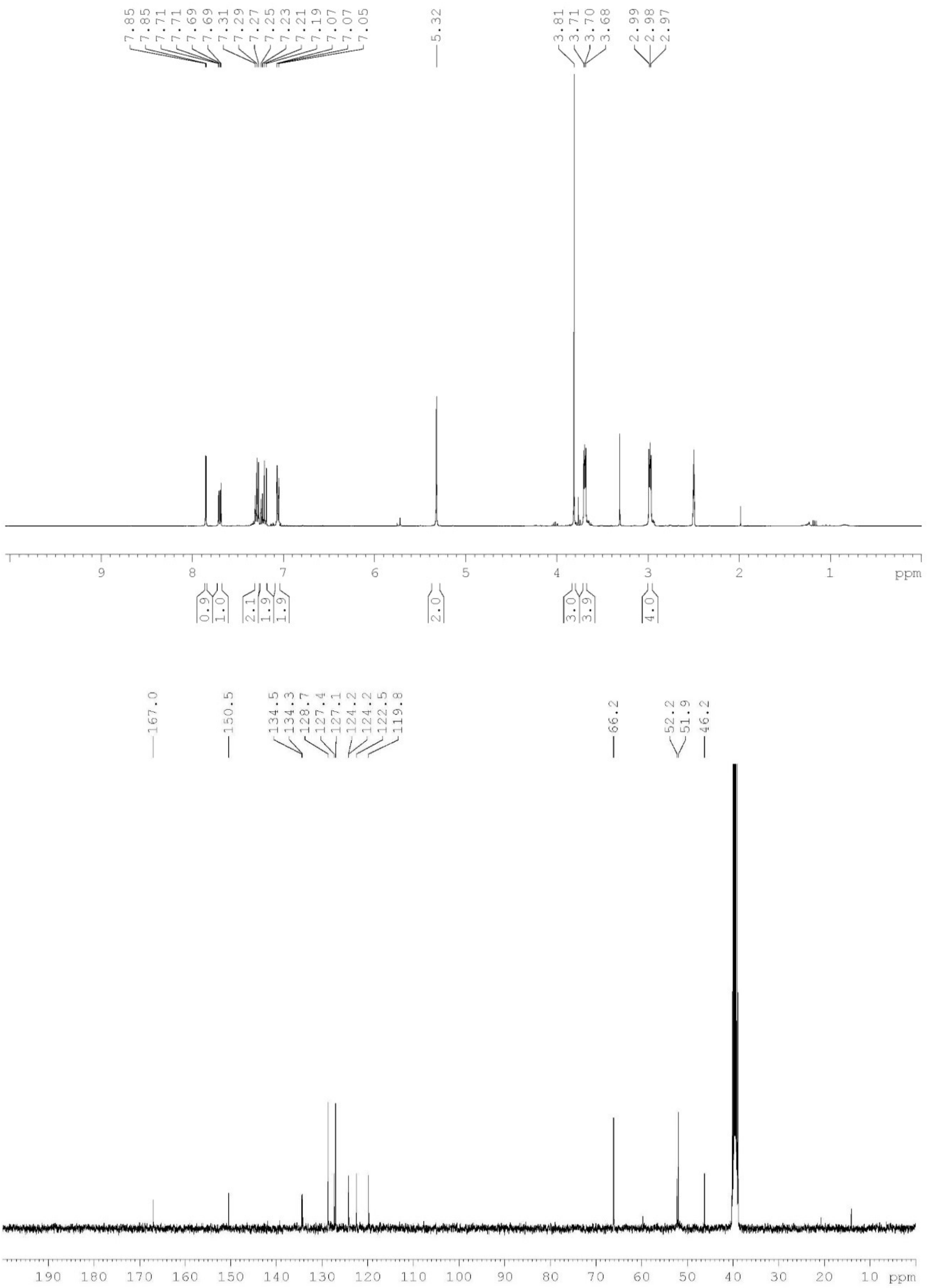

Figure S1: ^{1}H NMR and ^{13}C NMR spectra of **7a** (400/100 MHz; $(CD_3)_2SO$).

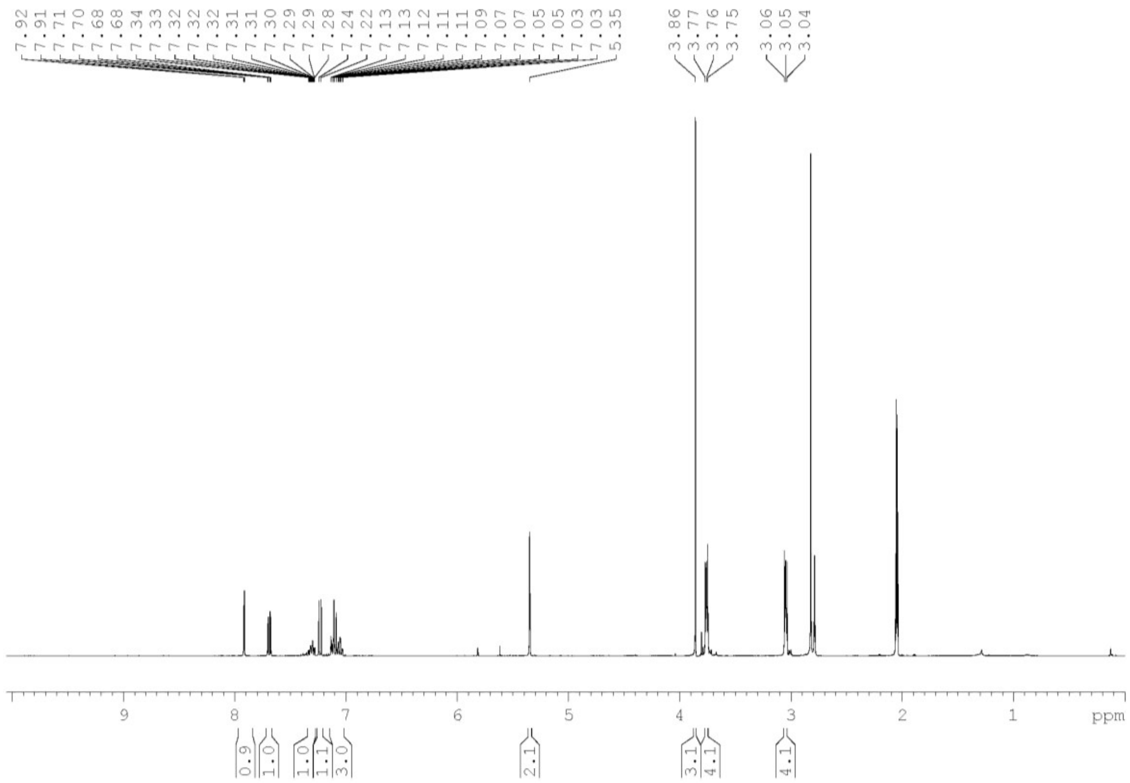

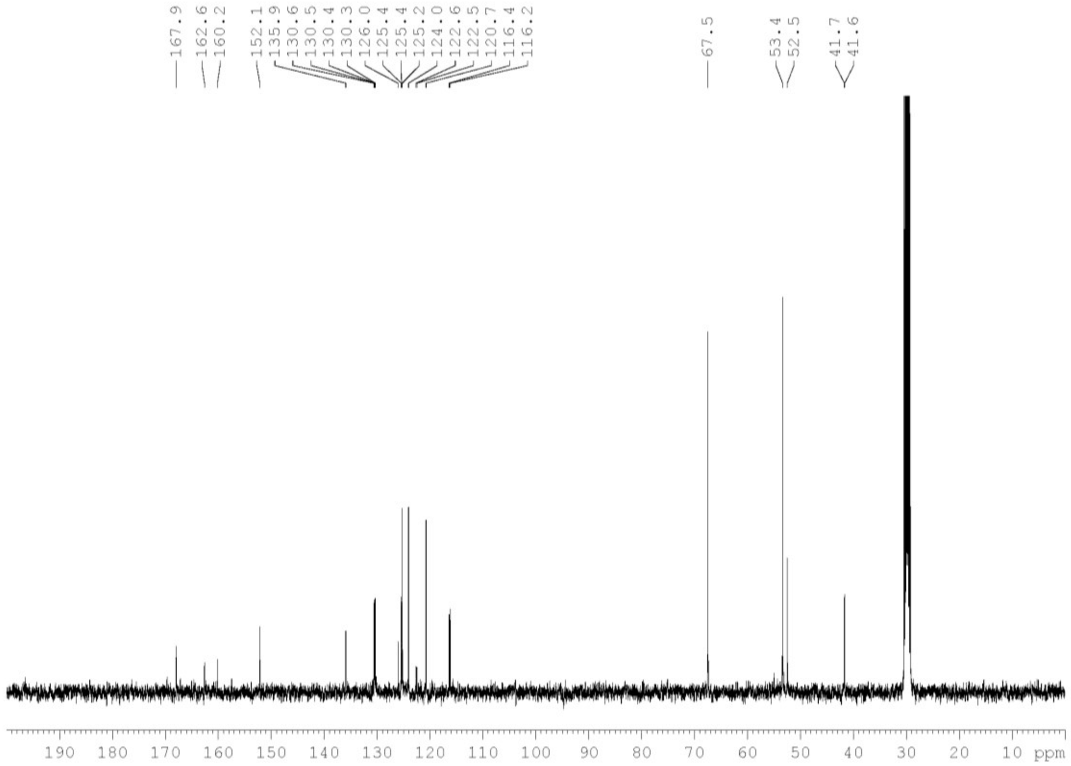

Figure S2: ^{1}H NMR and ^{13}C NMR spectra of **7b** (400/100 MHz; $(CD_3)_2CO$).

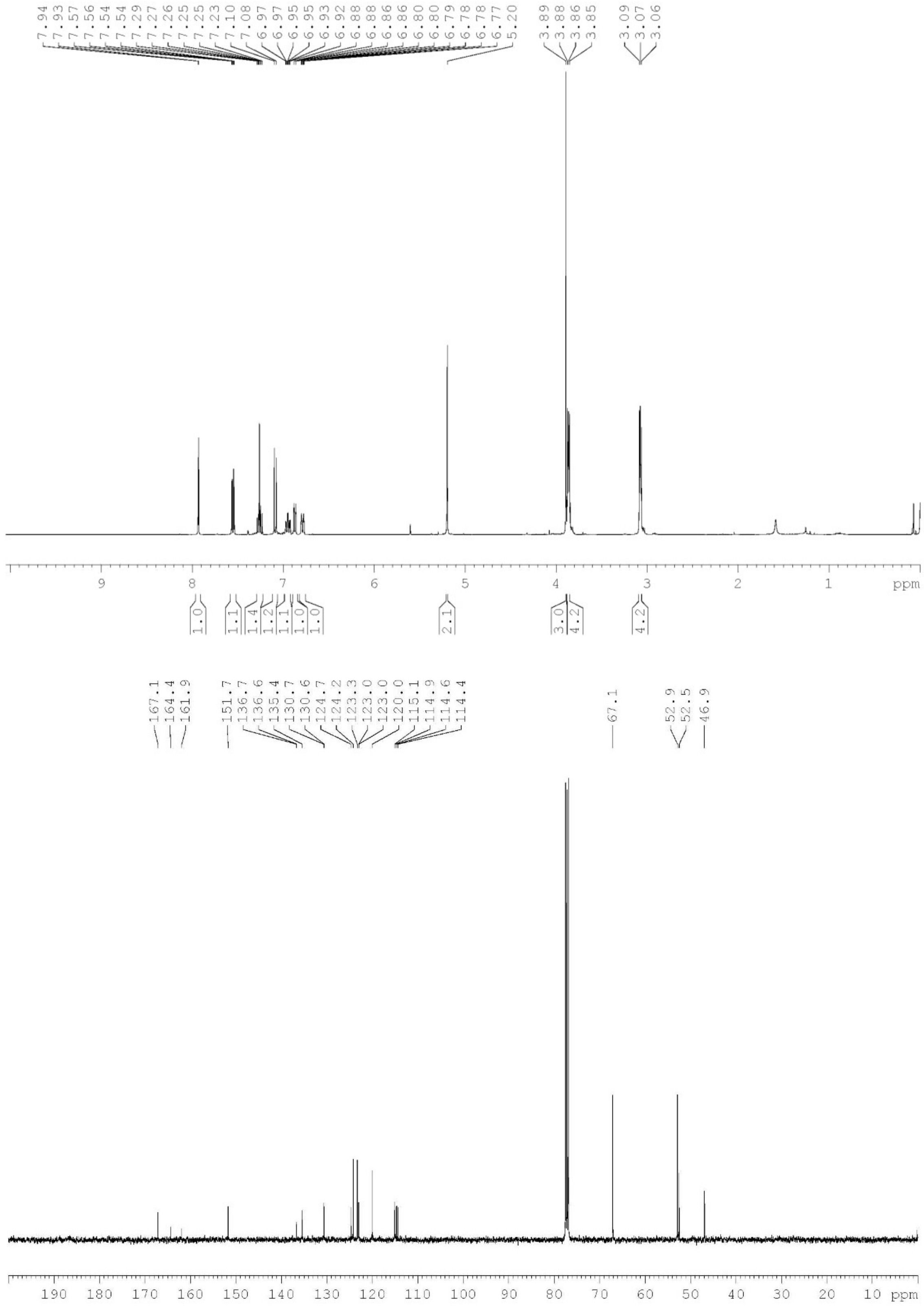

Figure S3: ^{1}H NMR and ^{13}C NMR spectra of **7c** (400/100 MHz; $CDCl_3$).

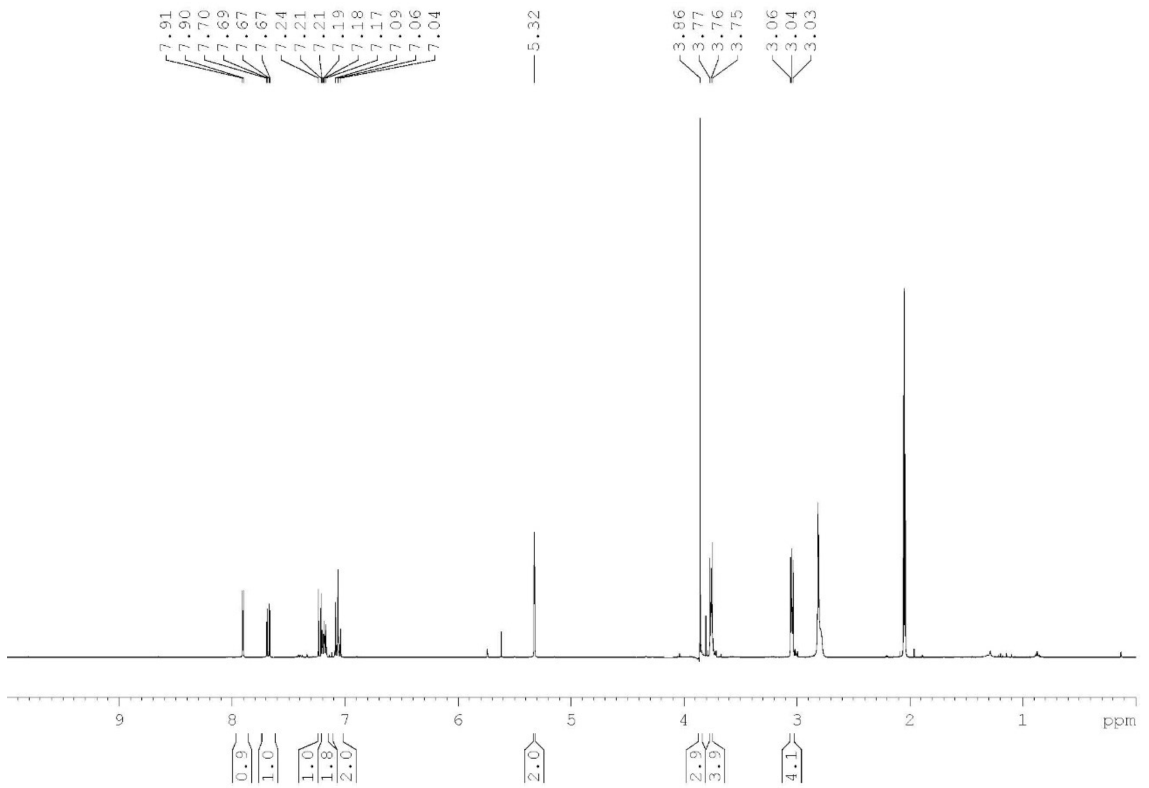

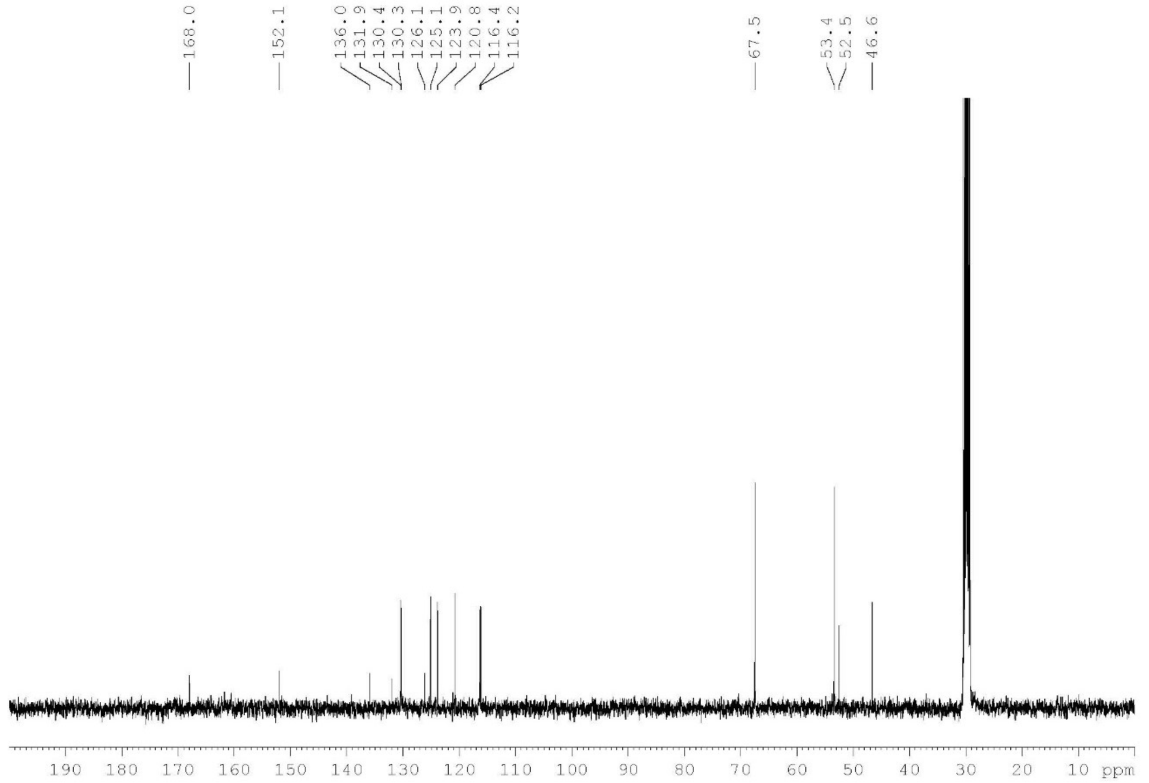

Figure S4: ^{1}H NMR and ^{13}C NMR spectra of **7d** (400/100 MHz; $(CD_3)_2CO$)

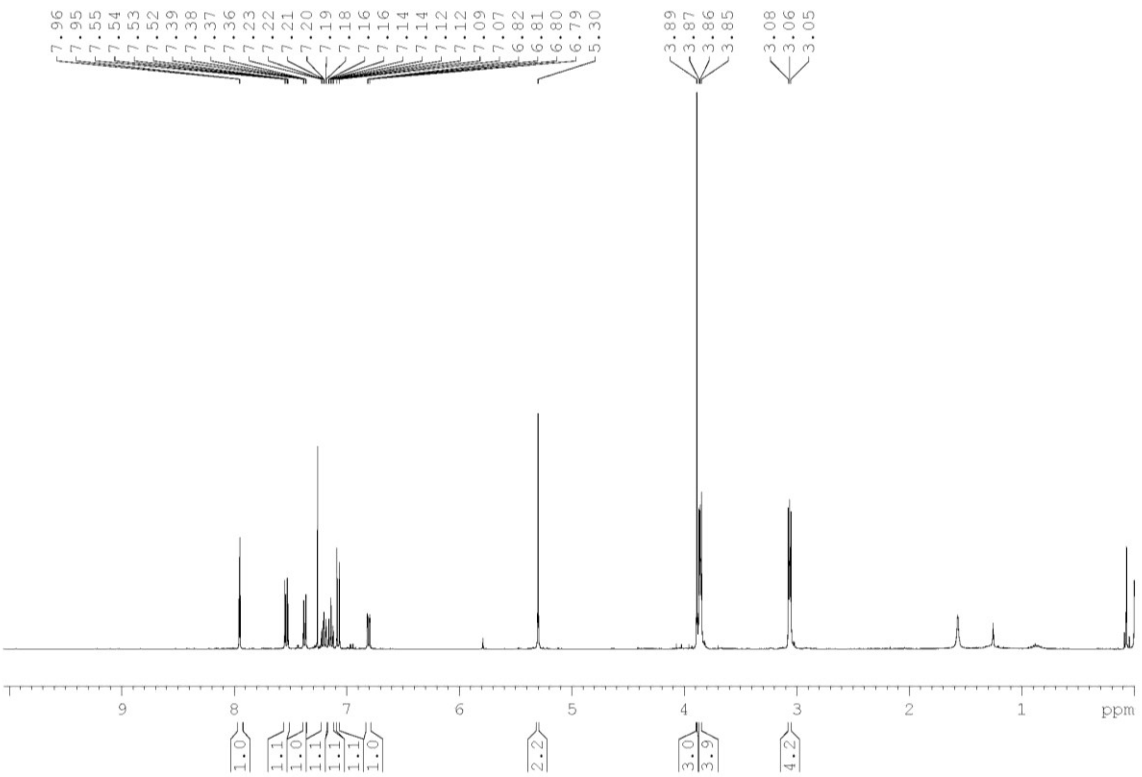

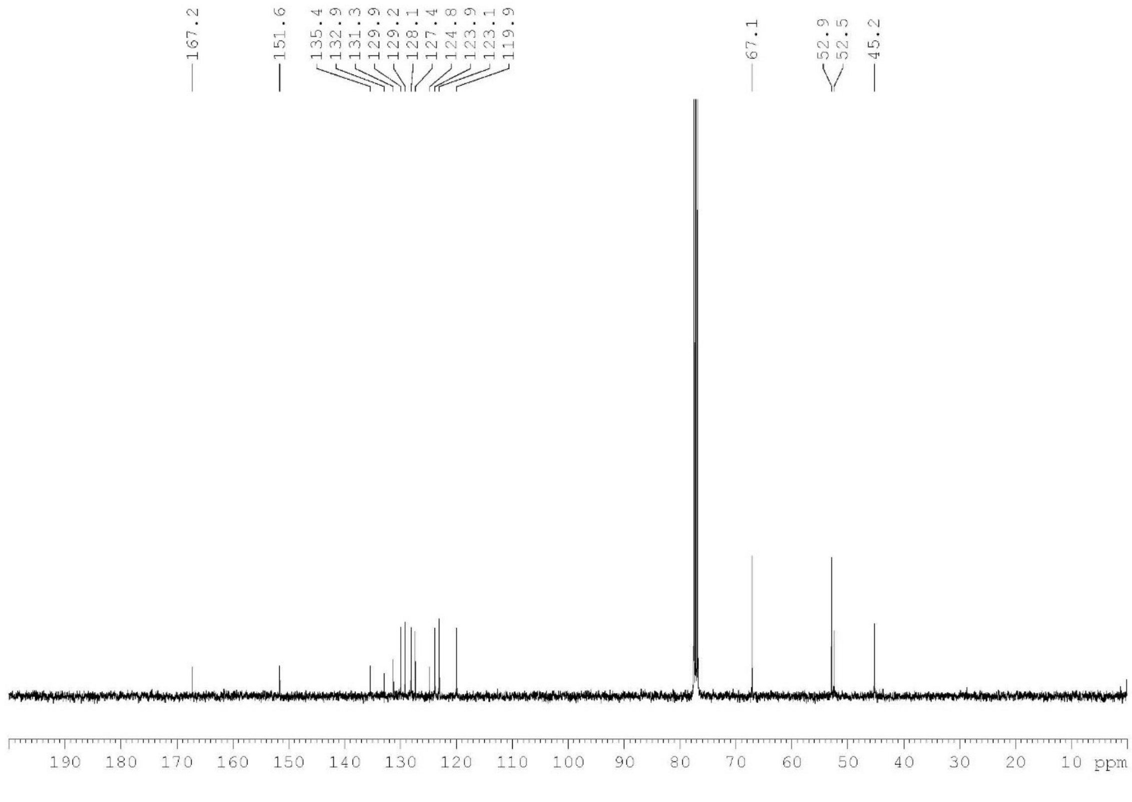

Figure S5: ^{1}H NMR and ^{13}C NMR spectra of **7e** (400/100 MHz; $CDCl_3$).

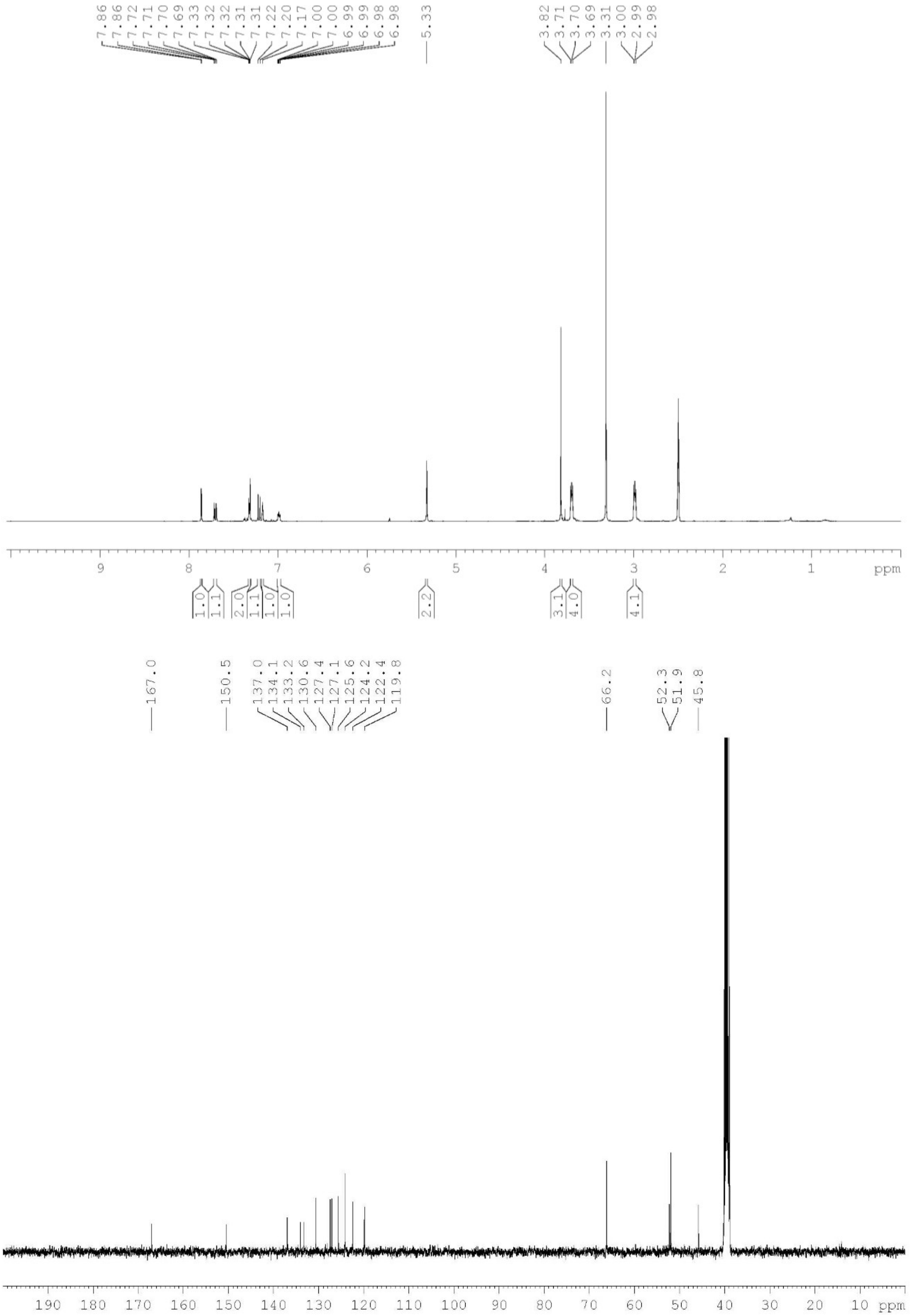

Figure S6: ^{1}H NMR and ^{13}C NMR spectra of **7f** (400/100 MHz; $(CD_3)_2SO$).

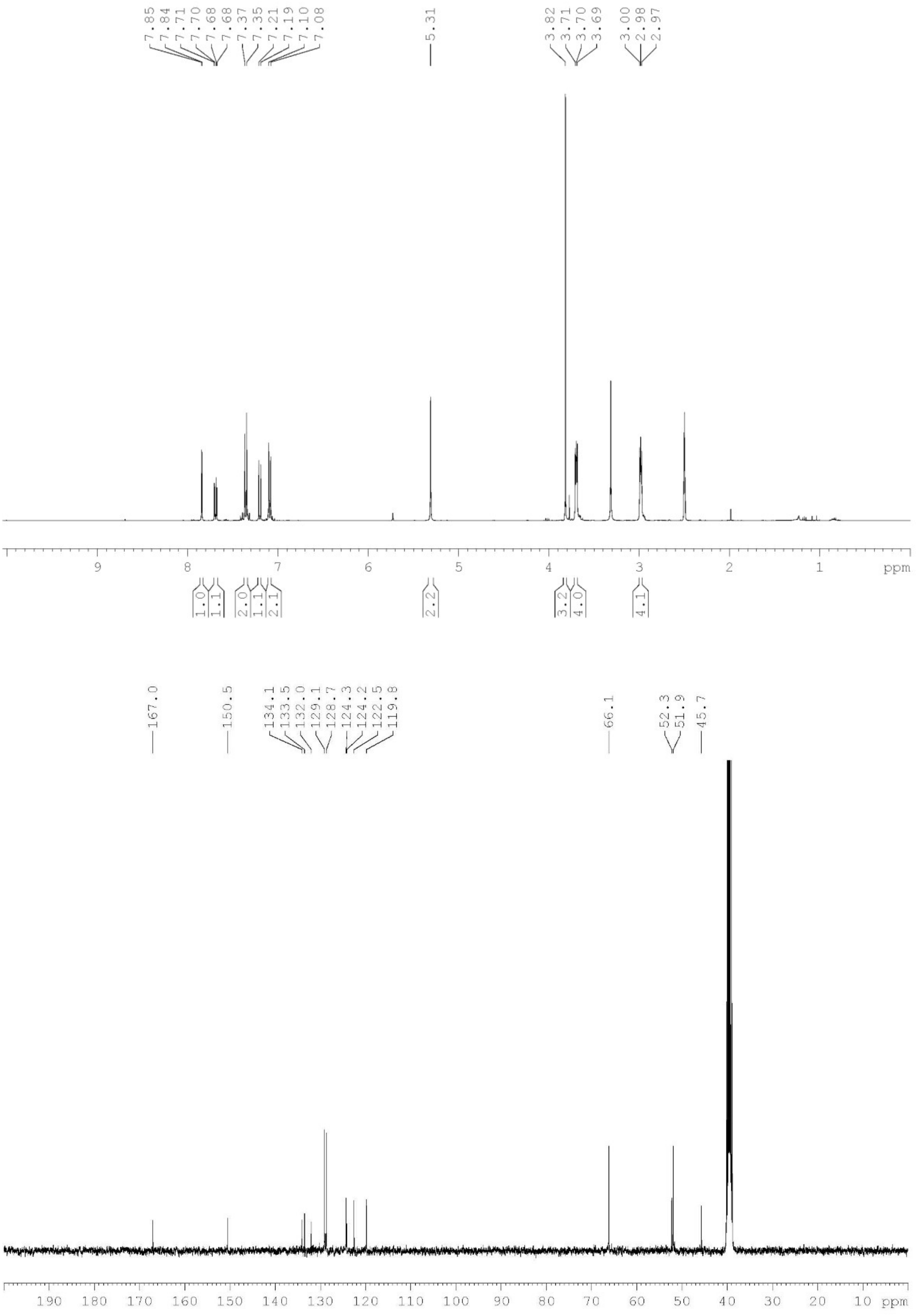

Figure S7: ^{1}H NMR and ^{13}C NMR spectra of **7g** (400/100 MHz; $(CD_3)_2SO$).

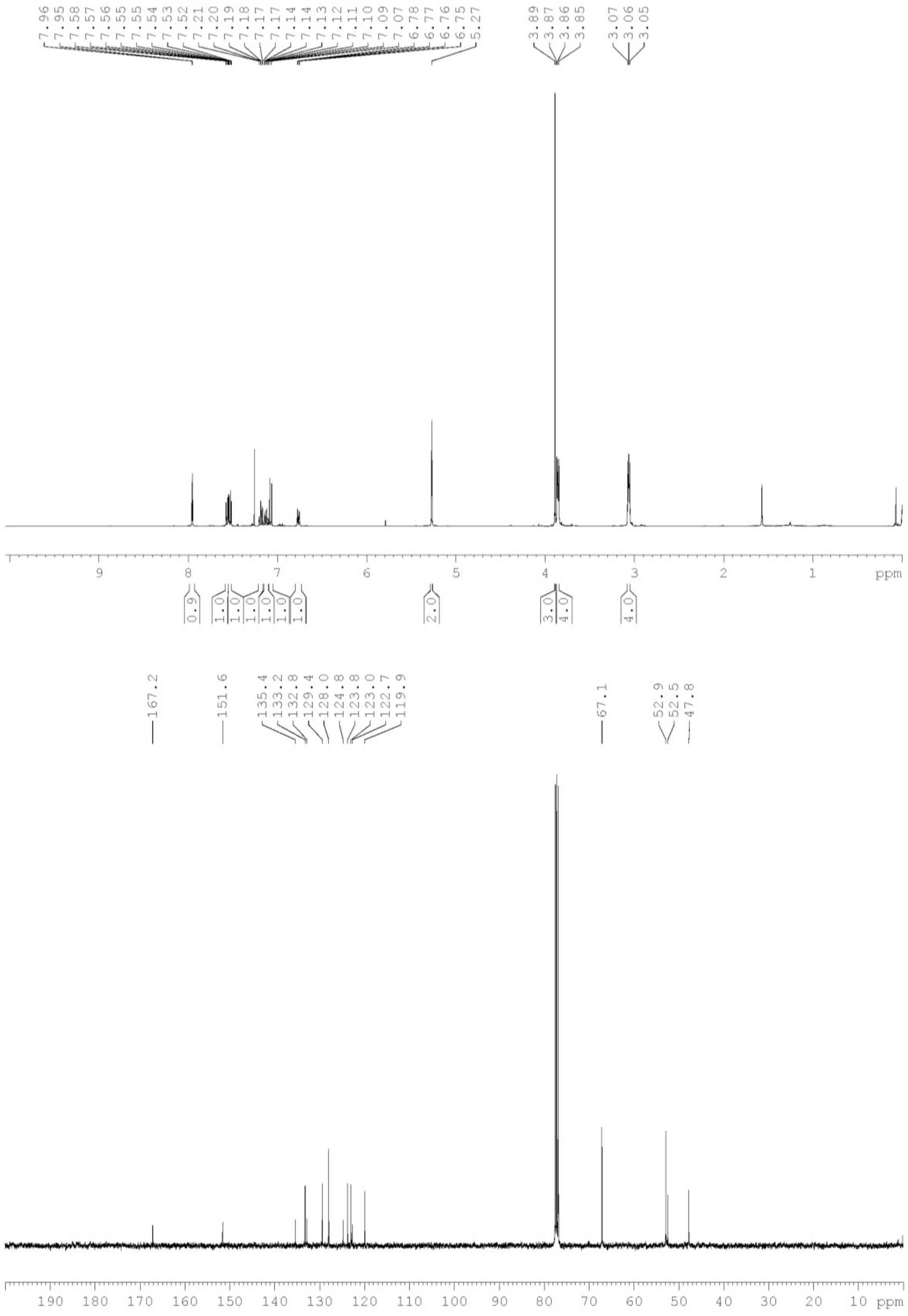

Figure S8: ^{1}H NMR and ^{13}C NMR spectra of **7h** (400/100 MHz; $CDCl_3$).

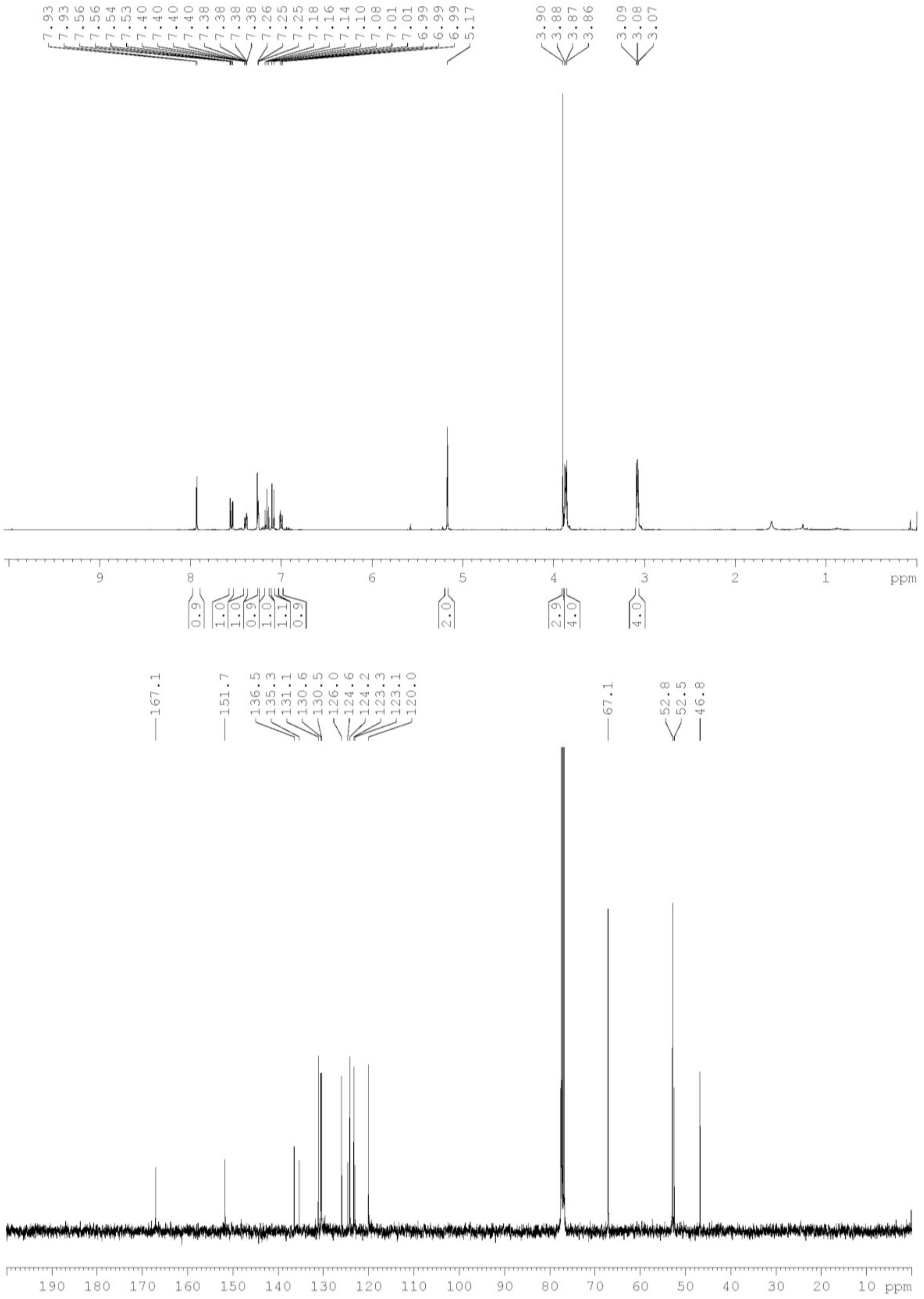

Figure S9: ^{1}H NMR and ^{13}C NMR spectra of **7i** (400/100 MHz; $CDCl_3$).

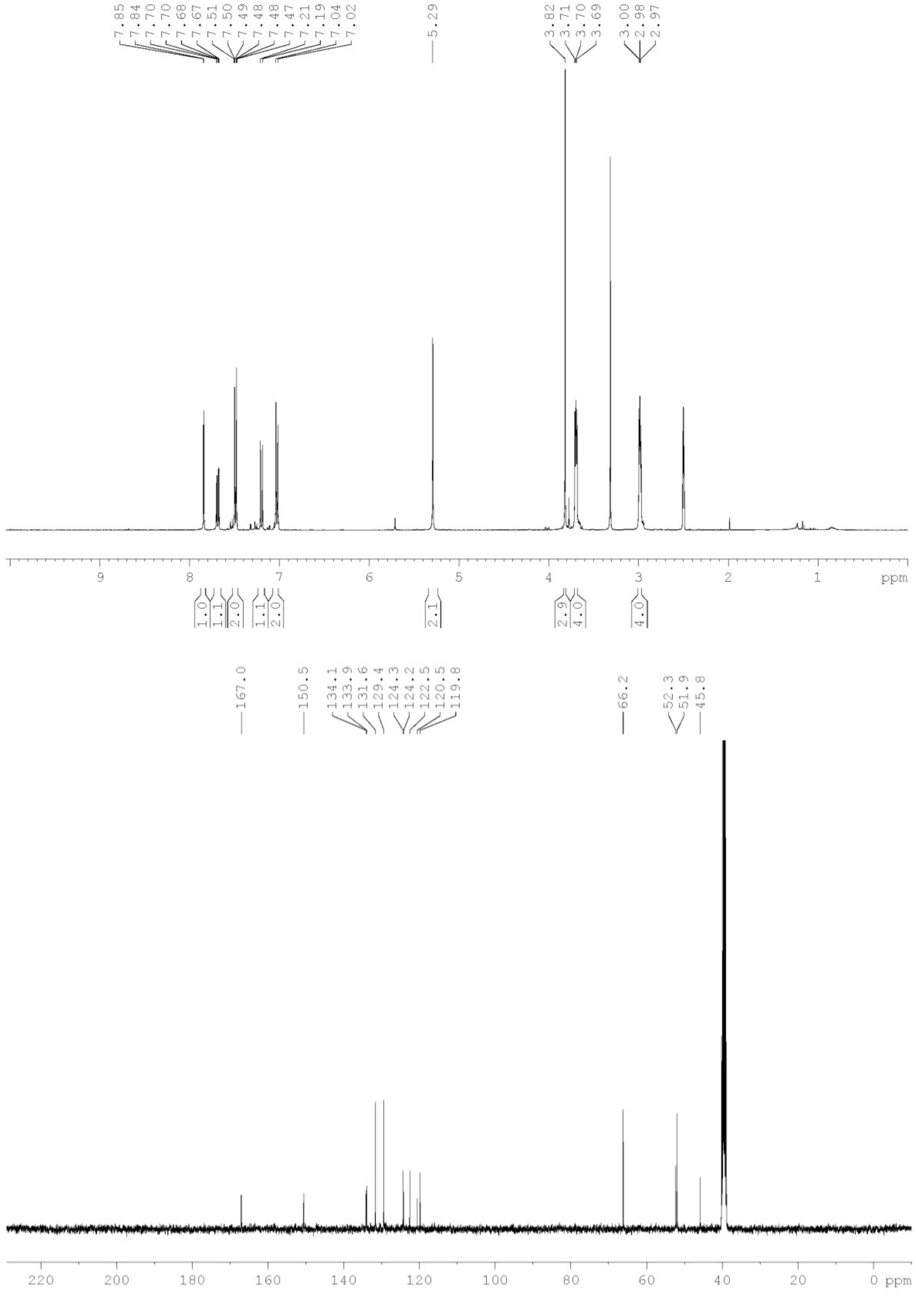

Figure S10: ^{1}H NMR and ^{13}C NMR spectra of **7j** (400/100 MHz; $(CD_3)_2SO$).

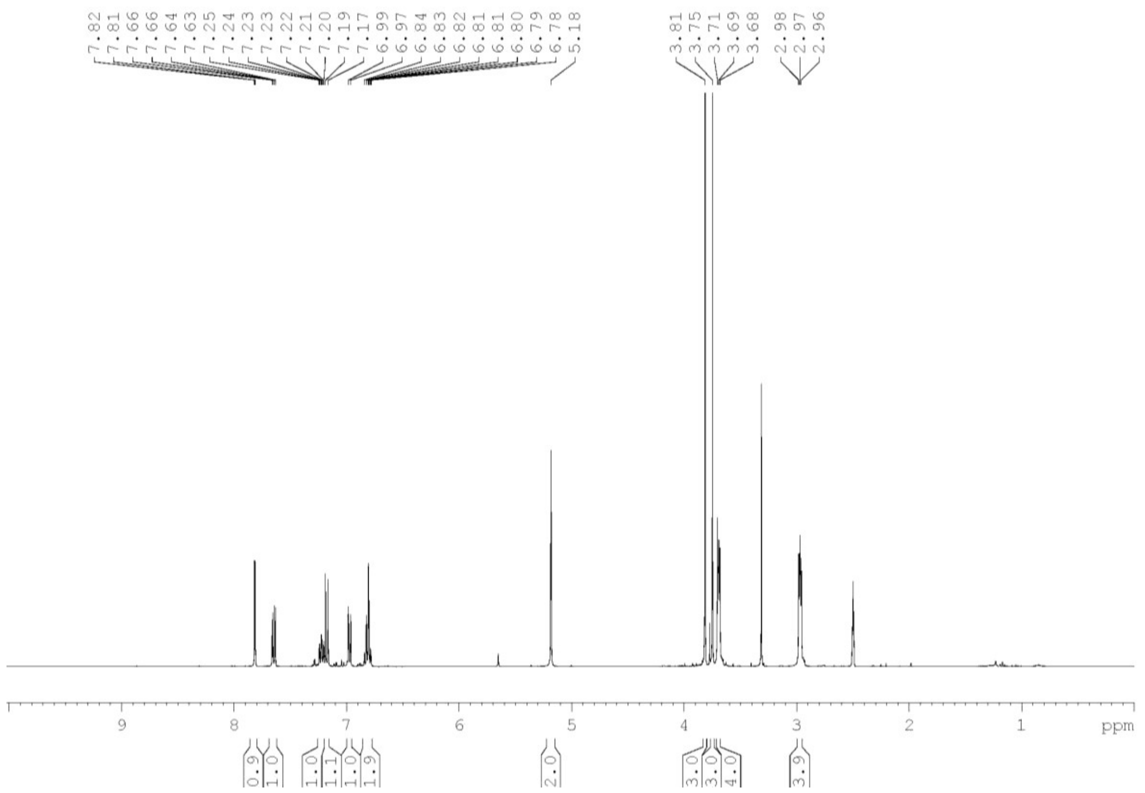

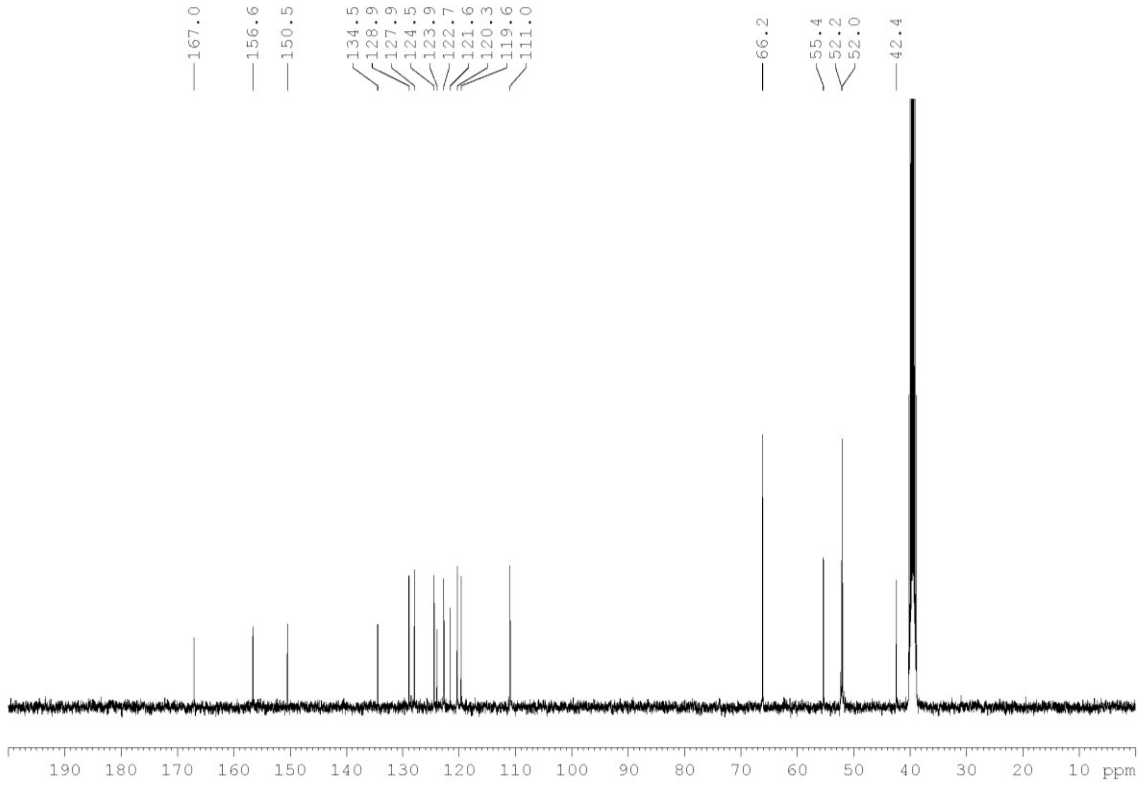

Figure S11: ^{1}H NMR and ^{13}C NMR spectra of **7k** (400/100 MHz; $(CD_3)_2SO$).

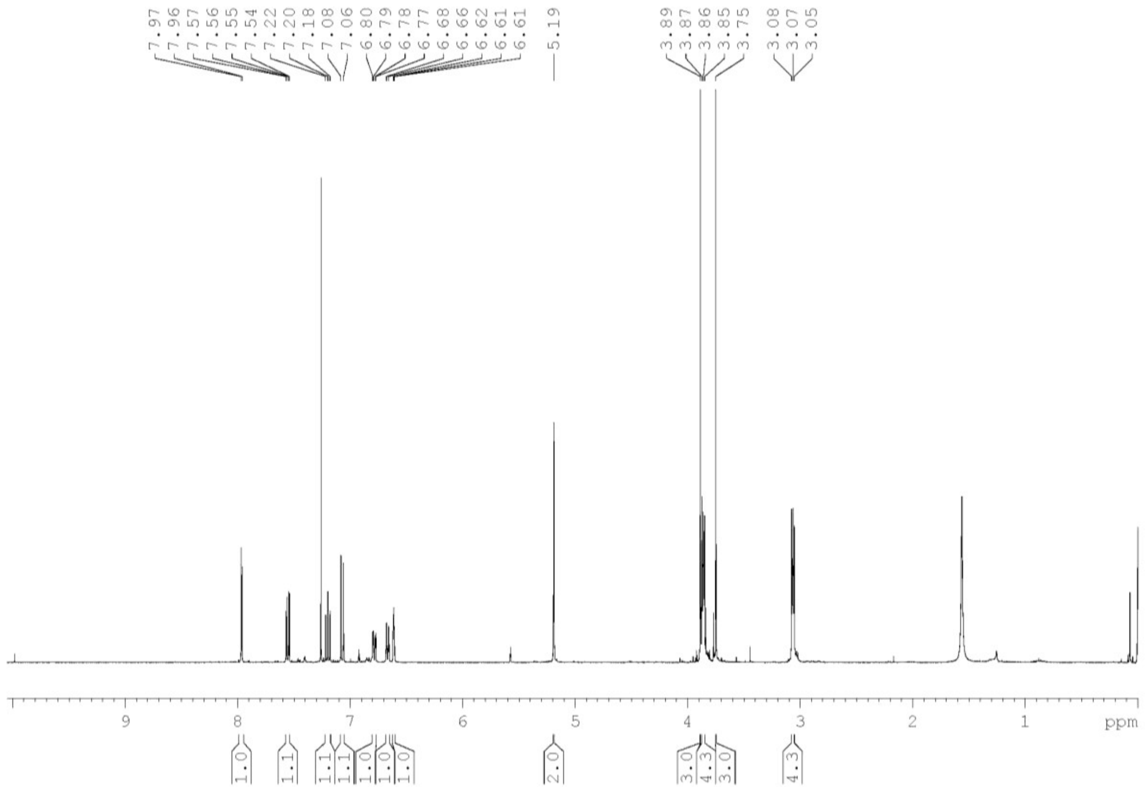

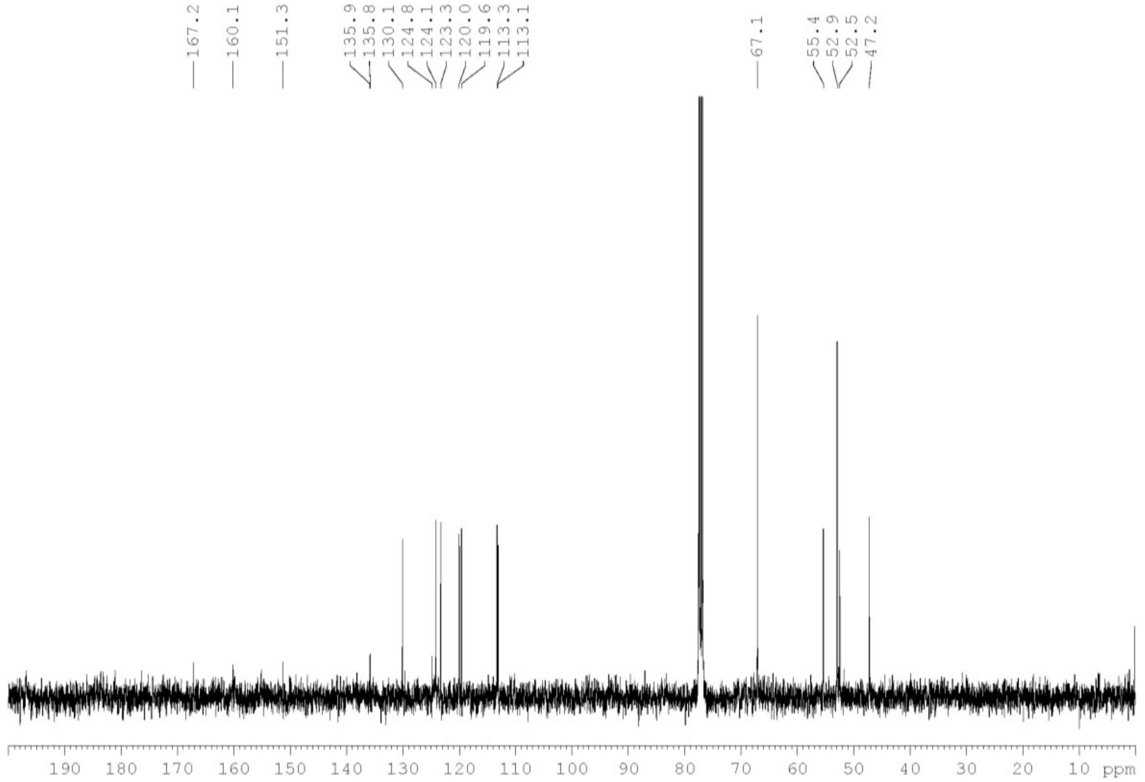

Figure S12: ^{1}H NMR and ^{13}C NMR spectra of **7l** (400/100 MHz; $CDCl_3$).

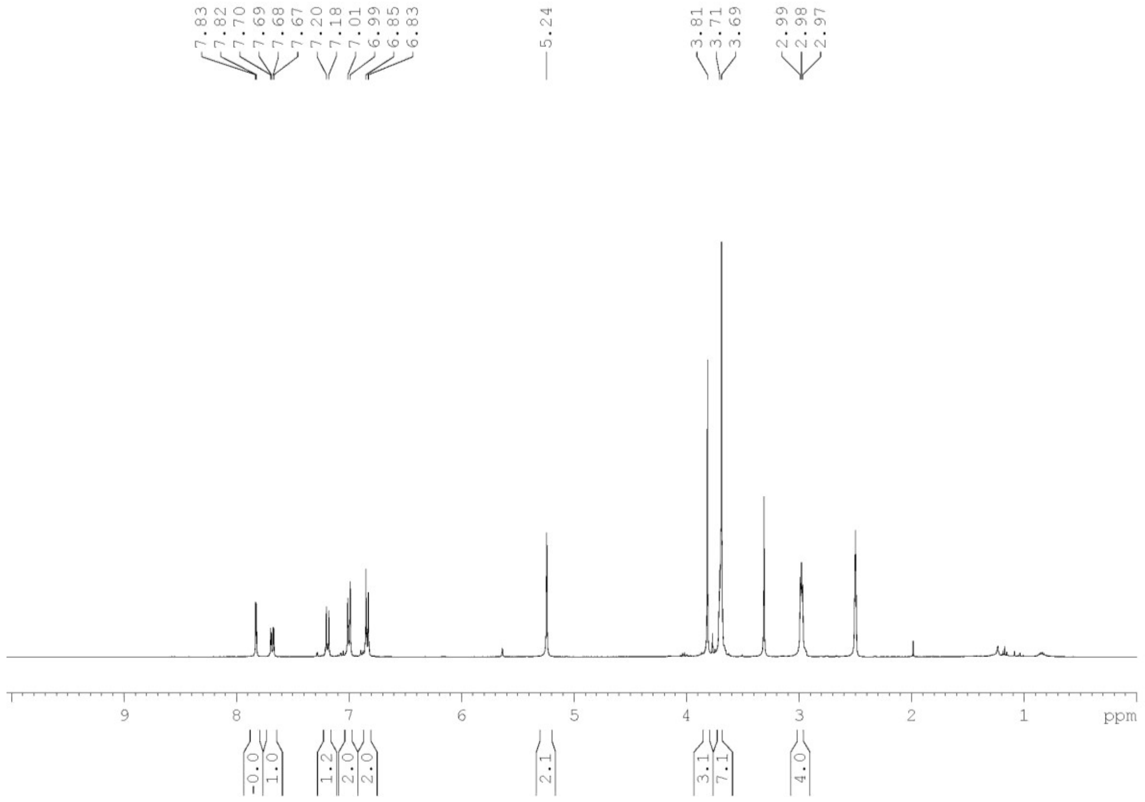

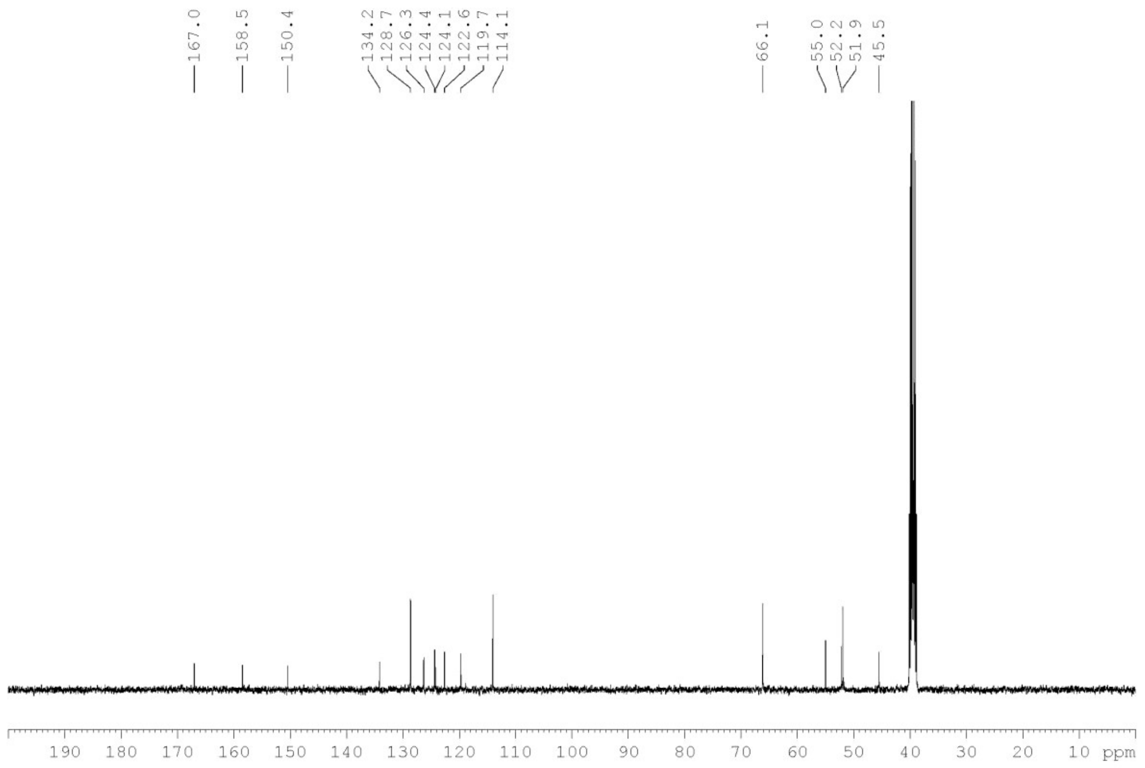

Figure S13: ^{1}H NMR and ^{13}C NMR spectra of **7m** (400/100 MHz; $(CD_3)_2SO$).

Table S1: Drug-likeness of *N*-nitrosylated benzylamines **7a-m** according to Lipinski's rule of five. All compounds adhere to the requirements (MW < 500, HBD < 5, HBA < 5, cLogP < 5).[25]

Benzylic Substitution of **7**	Molecular Weight	Hydrogen Bond Donors	Hydrogen Bond Acceptors	Calculated LogP
H (**a**)	355.39	0	7	2.804
2-F (**b**)	373.38	0	7	2.947
3-F (**c**)	373.38	0	7	2.947
4-F (**d**)	373.38	0	7	2.947
2-Cl (**e**)	389.84	0	7	3.517
3-Cl (**f**)	389.84	0	7	3.517
4-Cl (**g**)	389.84	0	7	3.517
2-Br (**h**)	434.29	0	7	3.667
3-Br (**i**)	434.29	0	7	3.667
4-Br (**j**)	434.29	0	7	3.667
2-OMe (**k**)	385.42	0	8	3.723
3-OMe (**l**)	385.42	0	8	3.723
4-OMe (**m**)	385.42	0	8	3.723
